# Trans-anethole attenuates streptozotocin-induced cognitive dysfunction by modulating oxidative stress and cholinergic activity

**DOI:** 10.3389/fphar.2026.1954658

**Published:** 2026-10-16

**Authors:** Shruti S. Mole, Prarambh S. R. Dwivedi, Wilona Loren Lobo, C. S. Shastry, Pukar Khanal

**Affiliations:** 1 Department of Pharmacology, NGSM Institute of Pharmaceutical Sciences (NGSMIPS), Nitte (Deemed to be University), Mangalore, India; 2 Silicon Script Sciences Pvt. Ltd., Ghorahi, Nepal

**Keywords:** Alzheimer’s disease, oxidative stress, PI3K-Akt signaling, streptozotocin, TNF signaling, trans-anethole

## Abstract

**Introduction:**

Cognitive impairment is a neurological disorder defined as an inability to learn, think, judge, remember, and make decisions. The primary cause of chronic, age- related cognitive decline is Alzheimer’s disease (AD), which is frequently studied as a representative model. The current therapies for AD provide only symptomatic relief, which necessitates the need for safer, multi-target treatments. Trans- anethole, the primary constituent of *Illicium verum*, *Pimpinella anisum*, and *Foeniculum vulgare*, is an aromatic molecule that possesses several pharmacological activities, including anti-inflammatory and antioxidant effects, which may have neuroprotective benefits.

**Methods:**

The current study integrated *in silico*, *in vitro*, and *in vivo* approaches to examine the neuroprotective potential of trans- anethole. *In silico* studies were carried out to find out the potential molecular targets and pathways associated with the effects of trans-anethole against AD- related cognitive dysfunction. Cytotoxicity, antioxidant activity, and cholinesterase inhibition were assessed *in vitro* on BV2 cells. Intracerebroventricular (ICV) administration of streptozotocin (STZ: 3 mg/kg) in rats induces an insulin-resistant brain state leading to progressive cognitive and memory impairment, a condition that closely relates to sporadic Alzheimer’s disease. ICV-STZ-induced Wistar rats were used to assess the effect of trans- anethole on AD-related cognitive dysfunction through behavioral, biochemical, and histopathological assessments.

**Results:**

A total of 174 common targets were identified, enriching pathways including apoptosis, AGE-RAGE, TNF, and PI3K- Akt signaling. Stable interactions were observed in molecular docking and molecular dynamics simulations. Trans-anethole demonstrates moderate cholinesterase inhibition and antioxidant activity in BV2 cells. Comparing STZ- treated rats to disease control, trans-anethole at doses of 100 mg/kg and 400 mg/kg significantly reduced lipid peroxidation, restored antioxidant enzyme levels, and attenuated cholinergic dysfunction. Histopathological findings showed qualitative preservation of hippocampal neuronal morphology.

**Discussion:**

Overall, these findings suggest that trans-anethole may represent a potential neuroprotective preclinical candidate for further investigation in Alzheimer’s disease-related cognitive dysfunction.

## Graphical Abstract

**Figure:**
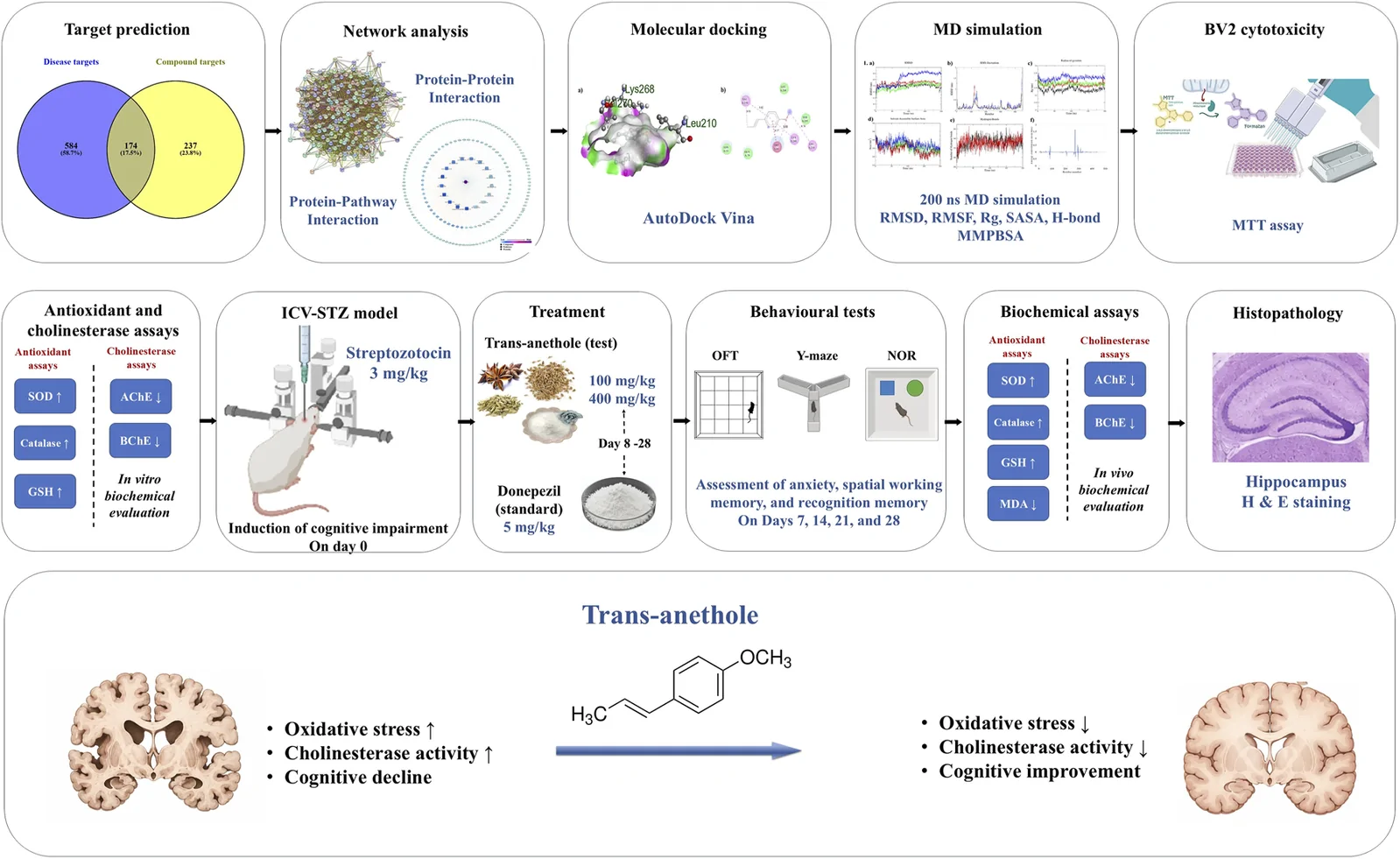

## 1 Introduction

Cognitive impairment is a symptom or a feature that can be caused by underlying neurological disorders (Omer et al., 2023). These cognitive impairments result from typical pathological alterations in the brain, such as the accumulation of amyloid-β (Aβ) plaques and neurofibrillary tangles composed of hyperphosphorylated tau protein, which are hallmarks of Alzheimer’s disease (AD). Even though a wide range of conditions contribute to cognitive impairment, AD is the primary cause of chronic, age-related cognitive decline and is frequently studied as a representative model (Alzheimer Association, 2026); it is a progressive neurodegenerative condition characterized by deterioration of cognitive function and is associated with the accumulation of these pathological hallmarks. Even though there are currently several medications available for treatment, including memantine, galantamine, rivastigmine, and donepezil, they only relieve symptoms and do not stop the course of the disease (Pathan, 2023). This makes it essential to identify novel drug molecules for the treatment of AD.

Plants have long been a major source of therapeutic agents, and several plant-derived compounds have also been reported to have neuroprotective effects in AD (Zaky et al., 2017; Banu and Das, 2025b). Hence, we identified an aromatic molecule, trans-anethole, possessing several pharmacological activities, such as antioxidant, anti-inflammatory, antibacterial, antifungal, anticonvulsant, antinociceptive, and anticancer properties. Trans-anethole, a non-toxic, most abundant isomer, can be obtained from various plants like *Illicium verum, Pimpinella anisum, and Foeniculum vulgare* (Conceição-Santos et al., 2025). It is also used in perfumery as well as the food, cosmetic, and medicinal industries because of its pleasant, herbal scent and finds extensive application in pharmaceutical products. Additionally, trans-anethole was shown to have a neuroprotective impact on cortical neuronal cells and an anti-amnesic effect in behavioral tasks. Recent reports suggest that trans-anethole may be beneficial in the treatment of neurodegenerative diseases (Chang et al., 2022).

Streptozotocin (STZ) is a neurotoxic glucosamine-nitrosourea compound obtained from *Streptomyces achromogenes* (Xie et al., 2025). It is used to produce a non-transgenic animal model of sporadic AD. STZ causes the development of brain insulin resistance, which starts a series of neuropathological events that resemble the characteristics of AD in humans (Shi et al., 2017; Wang et al., 2018; Osmanović Barilar et al., 2023). STZ injections administered intracerebroventricularly (ICV) are a well-known experimental model for sporadic Alzheimer’s disease. ICV-injected STZ results in cholinergic dysfunction, tau protein hyperphosphorylation, insulin receptor dysfunction, mitochondrial dysfunction, oxidative stress, and impaired energy metabolism, which are hallmarks of AD. A study reported that repeated ICV administration of STZ at a dose of 3 mg/kg induces cognitive impairment in rats, accompanied by alterations in oxidative stress and neuroinflammatory mediators (Rai et al., 2013; Lui and Tsao, 2024). Additionally, assessing the efficacy of trans-anethole in a streptozotocin-induced intracerebroventricular (ICV) model of cognitive impairment provides an experimental approach to investigate its potential effects on cognitive dysfunction and AD-related pathological alterations.

Alzheimer’s disease is associated with impairment of multiple cognitive domains, including amnestic deficits, executive function deficits, aphasia, agnosia, apraxia, and attention deficits. *In vivo* behavioral studies are widely used to evaluate cognitive dysfunction in experimental models of AD. Common behavioral paradigms include the Morris water maze, the novel object recognition test, the Barnes maze test, the open field test, fear conditioning, the Y maze, and the radial arm maze, which assess different aspects of learning, memory, recognition, spatial cognition, and exploratory activity (Wahl et al., 2017).

Therefore, the current investigation aimed to combine *in silico, in vitro*, and *in vivo* procedures to assess the potential of trans-anethole against AD. Computational pharmacology approaches such as network pharmacology, molecular docking, and dynamic simulations were used to identify targets modulated by trans-anethole. In addition, antioxidant, acetylcholinesterase inhibitory activity, and butyrylcholinesterase activity of trans-anethole were assessed in BV2 cell lines to elucidate its protective effects against oxidative stress and cholinergic dysfunction, two key pathological features of cognitive decline. Furthermore, behavioral assessments, including the open-field test, Y-maze, and novel object recognition test, were performed to evaluate anxiety, spatial working memory, and recognition memory, respectively, in the ICV-STZ-induced rat model.

## 2 Materials and methods

### 2.1 Materials

Fetal bovine serum (RM9955), HiPurA® RIPA Lysis Buffer (SKU: ML242), Dulbecco’s Modified Eagle’s Medium (DMEM)-High Glucose (SKU: AL251), Dimethyl sulfoxide (SKU: GRM5856), and MTT (3-(4,5-dimethylthiazol-2-yl)-2,5-diphenyltetrazolium bromide) reagent (SKU: RM1131) from HiMedia Laboratories Pvt. Ltd. Thane, Maharashtra). BV2 cells were obtained from the National Center for Cell Science (NCCS, Pune), and Streptozotocin (CAS: 18883-66-4) from S R Laboratories Pvt. Ltd (Mumbai). Trans-anethole (CAS RN-4180-23-8) from Tokyo Chemical Industry (India) Pvt. Ltd, Donepezil (CAS RN, 120011-70-3) from Yarrow Chem Products (Mumbai), Agappe total protein diagnostic kit (CAS No: 51013002) from Agappe Diagnostics Ltd. (Kerala), sodium carbonate (497-19-8), nitroblue tetrazolium (298-83-9), hydroxylamine hydrochloride (4131D 00250), hydrogen peroxide (4131D 00250) from LOBA CHEMIE PVT. LTD., 5, 5-dithiobis-(2-nitrobenzoyl acid)-DTNB (4131D 00250), trichloroacetic acid (TCA) (CAS: 76-03-9), thiobarbituric acid (CAS: 504-17-6) from S R Laboratories Pvt. Ltd (Mumbai), acetylthiocholine iodide (1866-15-5), butyrylthiocholine iodide (CAS RN: 1866-16-6) from Tokyo Chemical Industry (India) Pvt. Ltd.

### 2.2 *In silico* evaluation

#### 2.2.1 Prediction of drug-likeness, ADME properties, and biological spectra of trans-anethole

The physicochemical characteristics and drug-likeness metrics like molecular weight, polar surface area (PSA), molecular volume, pKa, drug-likeness score, hydrogen bond acceptors (HBA), blood-brain barrier (BBB) score, hydrogen bond donors (HBD), LogS, and LogP were assessed using the Molsoft (https://molsoft.com/mprop/) database. SwissADME (https://www.swissadme.ch/) was used to predict the ADME profile of trans-anethole (Safwat et al., 2021). The Pass Online (https://way2drug.com/PassOnline/; p > 0.7) database was used to identify the biological spectra of the compound (Druzhilovskiy et al., 2018; Khanal et al., 2022).

#### 2.2.2 Identification of targets

Trans-anethole targets were identified from DIGEP-Pred 2.0 (https://www.way2drug.com/digep-pred/; p_a_ > 0.7) and the Comparative Toxicogenomics Database (CTD; https://ctdbase.org/; interaction count ≥3) using previously retrieved Canonical SMILES from PubChem. The files were combined, and duplicates were removed to get the final drug targets (Lagunin et al., 2013; Davis et al., 2021; Khanal et al., 2022). The targets for the AD were obtained from databases like Online Mendelian Inheritance in Man (OMIM) (https://www.omim.org/), Therapeutic Target Database (TTD) (https://idrblab.net/ttd/) (Wang et al., 2020), GeneCards (https://www.genecards.org/), CTD (https://ctdbase.org/; inference score>50), and DisGeNET (https://www.disgenet.com/; GDA score>0.3) (Piñero et al., 2020), where Alzheimer’s Disease was used as a keyword. After this, the files were merged, and duplicates were removed to get the final disease targets.

Venny 2.1.0 (https://bioinfogp.cnb.csic.es/tools/venny/) was used to construct a Venn diagram to identify the common targets between trans-anethole and AD. To identify hub genes related to AD treatment, the intersecting targets were further investigated using computational pharmacology tools (Khanal et al., 2022).

#### 2.2.3 Gene set enrichment and gene ontology analysis

STRING (version 12.0, https://string-db.org/) was used to construct the protein-protein interaction (PPI) network of common targets and further visualized using Cytoscape (version 3.7.1). The common targets of AD and trans-anethole were used as input into the STRING database; *Homo sapiens* was selected as the organism to generate the interaction. To determine the pathways involved in AD, KEGG pathways were enriched. A protein-pathway interaction network for the 17 pathways was developed using Cytoscape to examine key pathways. The molecular function, cellular component, and biological processes of the common genes were predicted by Gene Ontology (GO) analysis (Dwivedi et al., 2021; Dwivedi and Shastry, 2023).

#### 2.2.4 Molecular docking

Trans-anethole’s binding affinity to the identified AD-related targets was assessed using molecular docking studies with AutoDock Vina. The 3D conformation of trans-anethole was obtained from the PubChem (https://pubchem.ncbi.nlm.nih.gov) database. Energy minimization was carried out using the MMFF94 force field by Discovery Studio Visualizer (BIOVIA Discovery Studio Visualizer; version 2021). The PDB IDs of proteins were obtained from the UniProt (https://www.uniprot.org/) database. Elimination of heteroatoms, pre-existing ligands, and water molecules from each target’s crystal structure, and addition of missing hydrogen atoms, were carried out using Discovery Studio Visualizer. Each target’s energy level was minimized using the MMFF94 force field. Homology modeling was done using the SWISS-MODEL tool (https://swissmodel.expasy.org/) using the FASTA sequences from UniProt. The ligand-protein docking was performed using AutoDock Vina. Trans-anethole and standard drugs (for AKT1: Sirolimus; APP: Curcumin; ATF4: Mecamylamine; BACE1: acetylcholine; BAD: Disopyramide; CASP3: Doxorubicin; CASP9: Pamidronic acid; CDK5: Trilaciclib; CYBB: Dextromethorphan; EIF2S1: Cyclosporic-A; FAS: Pyrazinamide; GAPDH: Reduced nicotinamide adenine dinucleotide (NADH); GRIN1: Memantine; IKBKB: Parthenolide; IL1B: Diacerein; IL6: Cholesterol; MAP2K1: Cholesterol; MAPK1: Erlotinib; MAPK3: Etoposide; MAPK9: Minocycline; NFKB1: Donepezil; NOS2: L-Arginine; PTGS2: Aspirin; RAF1: Acetylcholine; REAL: Dimethyl-fumarate; TNF: Budesonide; UQCRC1: Ubiquinone-2; VDAC1: Cannabidiol; XBP1: Valproic acid; AGER: Candesartan cilexetil; IDE: L-Alanine; and for NOS1: Cocaine) were docked against all known targets to find possible interactions; the binding energies and conformations of ligand-protein complexes were examined (Dwivedi et al., 2021; Khanal et al., 2023).

#### 2.2.5 Molecular dynamics simulation and MMPBSA analysis

Molecular dynamics (MD) simulations were performed for the selected protein-ligand complexes using GROMACS 2022.4. The CHARMM36 all-atom force field (July release) was used for protein parameterization, while ligand topology parameters were generated using CGenFF v4.6 through the CHARMM-GUI Ligand Reader and Modeller. Protein structures were prepared using the GROMACS pdb2gmx module, and protonation states were assigned based on predictions at pH 7.4 using H++/PROPKA. Each protein-ligand complex was placed in a cubic simulation box with a minimum padding of 1.0 nm from the box boundaries and solvated using the SPC water model. Na^+^ and Cl^−^ ions were added to neutralize the systems and achieve an ionic strength of 0.15 M. The resulting systems contained approximately 55,000-75,000 atoms. Energy minimization was performed using the steepest-descent algorithm for a maximum of 50,000 steps, until maximum force was below 1,000 kJ mol^-1^ nm^-1^. The minimized systems were subsequently equilibrated under NVT conditions for 100 ps at 300 K using the V-rescale thermostat (τT = 0.1 ps), followed by NPT equilibration for 100 ps at 1 bar using the Parrinello-Rahman barostat (τP = 2.0 ps; compressibility = 4.5 × 10^−5^ bar^-1^). A 2-fs integration timestep was employed, and covalent bonds involving hydrogen atoms were constrained using the LINCS algorithm (order = 4; one iteration). Long-range electrostatic interactions were calculated using the Particle Mesh Ewald (PME) method with a real-space cutoff of 1.2 nm, Fourier grid spacing of 0.16 nm, and fourth-order interpolation. Lennard-Jones interactions were switched off between 1.0 and 1.2 nm. Following equilibration, each complex underwent a 200 ns production MD simulation, with trajectory coordinates saved every 20 ps for subsequent analysis. The trajectories were analyzed for Root Mean Square Deviation (RMSD), Root Mean Square Fluctuation (RMSF), radius of gyration (Rg), Solvent Accessible Surface Area (SASA), and hydrogen-bond formation to characterize the structural and dynamic behavior of the protein-ligand complexes (Dwivedi et al., 2021; Dwivedi and Shastry, 2023).

Binding free energy and its key components, including electrostatic, van der Waals, and solvation energies, were assessed using the gmx_MMPBSA module based on Molecular Mechanics Poisson-Boltzmann Surface Area (MMPBSA) analysis. A total of 100 frames were selected from 10,000 simulation frames at an interval of 100, and Poisson-Boltzmann calculations were performed using Sander’s built-in PBSA solver, and results were analyzed using the MMPBSA_ana module (Khanal et al., 2022).

#### 2.2.6 Principal component analysis and free energy landscape

The equilibrated MD trajectory was subjected to Principal Component Analysis (PCA) in order to examine the dominating collective movements of the protein-ligand combination. Periodic boundary conditions were eliminated before analysis began, and overall translational and rotational motions were eliminated by fitting the trajectory to the reference structure using least-squares fitting. The eigenvectors and associated eigenvalues were then obtained by diagonalizing a covariance matrix of atomic positional fluctuations. The eigenvalues show the amplitude of motion along each primary component, whereas the eigenvectors define the direction of aggregate atomic motions. The GROMACS gmx covar function was used to produce the covariance matrix and eigenvectors. gmx anaeig was used to project the trajectory along specific principal components to examine time-dependent conformational changes (Jain et al., 2010; Maisuradze et al., 2010).

During the MD simulation, the energy minima and conformational stability of the protein-ligand complex were investigated using Free Energy Landscape (FEL) analysis. The first two principal components, PC1 and PC2, were taken from the equilibrated trajectory using principal component analysis (PCA). A free energy surface was constructed using the trajectory projections along these components. The gmx_sham utility, which computes free energy as a function of PC1 and PC2 coordinates, was used to create the Gibbs free energy landscape. By identifying stable conformational states and energy basins, the resulting energy map shed light on the complex’s dynamic behavior and structural changes (Papaleo et al., 2009; David and Jacobs, 2014).

### 2.3 Experimental pharmacology

#### 2.3.1 *In vitro* study

##### 2.3.1.1 Maintenance and culture of BV2 cells

BV2 murine microglial cells were used as an *in-vitro* cellular model to evaluate the cellular antioxidant and cholinesterase-associated effects of trans-anethole. BV-2 cells were maintained in Dulbecco’s Modified Eagle Medium-High Glucose (DMEM-HG) supplemented with 10% FBS and 1% antibiotic-antimycotic. The cells were cultivated in a humidified environment with 5% CO_2_ at 37 °C. To assess the cytotoxicity, antioxidant, and cholinesterase activities, BV-2 cells were subjected to different doses of trans-anethole and donepezil; donepezil acts primarily as a reversible, highly specific inhibitor of the enzyme acetylcholinesterase, raising acetylcholine levels in brain synapses to support memory and cognition (Kumar et al., 2023; Li et al., 2021; Banu and Das, 2025a; Vilela et al., 2025).

##### 2.3.1.2 Cell cytotoxicity using MTT assay

The cells were seeded into a 96-well plate and left to adhere overnight. Following 24 h, cells were exposed to trans-anethole and donepezil at varying concentrations (1 nM, 10 nM, 100 nM, 1 μM, 10 μM, and 100 µM) along with a non-treated control for 24 h. Upon completion of treatment, the cells were washed, and freshly prepared MTT reagent (5 mg/mL) was added to each well and incubated for 4 h to complete the reaction. Upon completion of incubation, formazan crystals were visualized, and the cells were washed with PBS. Following this, 100 µL of DMSO was added and incubated for 1 h, followed by gentle shaking to solubilize the crystals. An ELISA multi-scan microplate reader was used to measure the absorbance at 570 nm (Fu et al., 2017; Gutiérrez et al., 2017).

The following formula was used to determine cell viability as a percentage in relation to the control:

$$Cell\;viability\;\left(\%\right)=\frac{Absorbance\;of\;test}{Absorbance\;of\;blank}\times 100$$

##### 2.3.1.3 Antioxidant, acetylcholinesterase (AChE), and butyrylcholinesterase (BChE) potential of trans-anethole in BV2 cells

The cells were seeded into a 30 mm dish and left to attach. After 24 h the cells were exposed to various drug concentrations (1 nM, 10 nM, 100 nM, 1 μM, 10 μM, and 100 µM) for 24 h. After 24 h, the cells were washed with lysis buffer, followed by the addition of 500 µL of lysis buffer, and the cells were collected. The collected cells were centrifuged at 10,000 rpm for 10 min at 4 °C, and the resulting supernatant was collected and used for further antioxidant procedures (Sigmaaldrich, 2026; Cobourne-Duval et al., 2016).

To evaluate the antioxidant properties of the test and standard drug, we assessed various antioxidant enzymes. The nitroblue tetrazolium (NBT) reduction inhibition method was carried out to evaluate the inhibition of NBT reduction by superoxide radicals at 560 nm. The GSH levels were analyzed using Ellman’s method, where the sample was deproteinized and treated with DTNB, which results in yellow TNB, measured at 412 nm. The catalase levels were measured as decomposed hydrogen peroxide (H_2_O_2_), measured at 240 nm (Akhtar et al., 2021).

Acetylcholinesterase and butyrylcholinesterase activities were determined by Ellman’s colorimetric method. The reaction mixture contained cell lysates of 24-hour-treated BV2 cells, DTNB, sodium phosphate buffer, and the sample treated with acetylthiocholine iodide and butyrylthiocholine iodide, and then the absorbance was measured at 412 nm (Kumar et al., 2008).

#### 2.3.2 *In vivo* evaluation

##### 2.3.2.1 Procurement and maintenance of animals

The study was approved by the Institutional Animal Ethics Committee, under approval number NGSMIPS/IAEC/APR-2025/515. Adult Wistar rats (n = 6 per group) of either sex weighing 150-200 g were procured from NUCARE animal house, Deralakatte, Mangalore. Rats were housed in a 22.5 cm × 37.5 cm polyacrylic cage with typical living conditions (room temperature and humidity, 24-27 °C and 60%-65%, respectively) and a 12-h light/dark cycle. Water and food in the form of dry pellets were provided for the rats.

##### 2.3.2.2 Groups and treatment schedule with test and standard drug, randomization, allocation, blinding, and exclusion criteria

Three distinct experimental cohorts were used to remove learning bias and behavioral test interference. Adult Wistar rats of either sex weighing 150-200 g were randomly divided into 5 groups (n = 6 per group) for each cohort. The rats were assigned to groups at random: vehicle, disease, standard, low-dose test, and high-dose group. The standard group received donepezil at a dose of 5 mg/kg orally, the test low-dose group received 100 mg/kg, and the test high-dose group received 400 mg/kg (INCHEM, 2026; Marinov and Valcheva-Kuzmanova, 2015), orally for 21 days, from day 8 to day 28. Trans-anethole’s safety profile was determined using previously published studies, which show that dosages up to 2000 mg/kg body weight are safe (rats). This method enhances internal validity and confirms that the observed biochemical changes result from treatment rather than stress-related effects.

Prior to the beginning of the experiment, body weights of all animals were recorded, and the animals were distributed into five groups with similar mean body weights so as to minimize possible differences in baseline body weight between groups. After grouping, the groups were designated as vehicle control, disease control, standard treatment, test low-dose, and test high-dose groups. Treatments were administered by a person who knew the group assignment. Behavioral assessments (OFT, Y-maze, and NOR) were analyzed by an investigator who was not blinded to treatment allocation. Similarly, histopathological evaluations were done with knowledge of treatment assignment. Animals that died during or after surgery were excluded from further analysis.

##### 2.3.2.3 Intracerebroventricular administration of streptozotocin using a stereotaxic apparatus

All surgical instruments were autoclaved, and the Hamilton syringe was cleaned by ejecting and drawing up nano-pure water repeatedly for 1 min. The procedure was repeated with 70% ethanol. Rats were subjected to anesthetization intraperitoneally with ketamine and xylazine (80 mg/kg and 10 mg/kg, respectively). After making sure that the animals were under anesthesia and had lost spontaneous movements, their scalp was shaved and made ready for the operation. To reveal the skull, a precise medial incision was made, and the bregma was identified. The precise stereotaxic coordinates, medial-lateral (ML), anterior-posterior (AP), and dorsal-ventral (DV), were identified. The lateral ventricular entry point was determined by going 0.8 mm posterior from the bregma, 1.4 mm lateral from the sagittal suture, and 3.6 mm vertically from the skull bone (Moreira-Silva et al., 2019). With the aid of a drill, a small spherical hole was produced over the marked coordinates. A single unilateral administration of Streptozotocin 3 mg/kg (More et al., 2026)**;** and the citrate buffer (for the vehicle group) was done through the punctured points with a Hamilton (2 µL) injector. After removing the animal from the apparatus, the hole was sealed with dental cement and a fixer. To prevent dry eyes, saline was applied, and Neosporin, an antiseptic, was used to protect against infections. After the surgical procedure, recovery of the animals was observed for 7 days (Tamam et al., 2023).

##### 2.3.2.4 Behavioral studies

For the behavioral assessment, the animals were trained two to three times before the surgery. The baseline reading was taken on the day of surgery. The behavioral assessments were carried out on 7, 14, 21, and 28 days after a 7-day recovery period. To remove learning bias and behavioral test interference, three distinct experimental cohorts were used.

###### 2.3.2.4.1 Cohort I: open field test (OFT)

OFT is a well-known task for evaluating anxiety-like behavior, which was used to examine anxiety in the models of Alzheimer’s disease. The test apparatus consists of a square arena with squares dividing the floor into sections and tall surrounding walls that stop the animal from escaping. Animals were subjected to the arena for 5 min and allowed to explore the area freely. The number of center crossings that each animal made was used to assess anxiety-like behavior (Pentkowski et al., 2021; Campos et al., 2023).

###### 2.3.2.4.2 Cohort II: spatial Y-maze memory test

Spontaneous alternation behavior was recorded using Y-maze equipment to study spatial working memory. A maze consisting of three arms, 120° apart, measuring 50 × 10 × 20 cm^3^. For 8 min, each rat was positioned at the end of an arm and given unrestricted access to the maze. Entry was deemed complete when the rat’s tail was completely contained within the arm. After every test, a 10% ethanol solution was used to clean the equipment and then patted dry with a paper towel. Visual records were made of the total number of arm entries (N) and the order of entries. Alternation behavior was described as making consecutive entries into each of the three arms (Shi et al., 2017). The following formula was used to assess the spontaneous alternation in behavior:

$$\text{Spontaneous alternation in behavior}=\frac{Total\;number\;of\;alterations}{\left(N-2\right)}\times 100$$

###### 2.3.2.4.3 Cohort III: novel object recognition test

The behavioral assay involved three phases, i.e., habituation, sample, and test. In the habituation phase, rats were introduced to an open field arena, explored for 5 min, and then returned to their home cage. In the sample phase, they were subjected to exploring two identical objects for 5 min, and in the test phase, they were tested to explore familiar and novel objects. For 5 min, rats were given free access to investigate both items. In the sample and test phases, the total time spent exploring the objects during the first 5 minutes was measured. The following actions were considered as item exploration: licking, smelling, contacting the object with the front legs or nose, and aiming the nose at an object less than 1 cm away. The discrimination index (DI), which measures the ratio of the time spent exploring the new object (T_new_) to the total time spent exploring the familiar object (T_familiar_), was used to determine the animal’s preference for the novel object (Ravelli et al., 2016; Drayson et al., 2023).

$$DI=\frac{\left(T_{new}-T_{familiar}\right)}{\left(T_{new}+T_{familiar}\right)}$$

It ranges from +1 to −1. The positive value of DI (**+1)** indicates a strong preference for novel objects (good memory). D = **0** shows no preference (memory impaired), and a negative DI value shows time spent with a familiar object (poor memory).

##### 2.3.2.5 Biochemical analysis

###### 2.3.2.5.1 Preparation of brain homogenate

On day 29, the animals were sacrificed with an overdose of isoflurane. The scalp was incised using a surgical blade, and the brain was carefully removed on an ice-filled plate. Then it was rinsed with cold phosphate buffer, sonicated, and homogenized in 10% w/v ice-cold phosphate-buffered saline (pH 7.4) and centrifuged for 20 min at 10,000 g at 4 °C to get the supernatant. Stored in the homogenized tissue supernatant at −20 °C for further biochemical analysis (Kumar et al., 2008).

###### 2.3.2.5.2 Estimation of total protein

Total protein content was assessed using the Agappe diagnostic kit (CAS No: 51013002). Bovine serum albumin served as the standard. The total protein analysis was performed as per the procedure provided in the kit, where 10 µL of supernatant and 500 µL of reagent were mixed and incubated for 10 min, and then the reading was taken.

###### 2.3.2.5.3 Superoxide dismutase (SOD)

SOD activity is based on the principle of its potential to suppress the reduction of nitroblue tetrazolium (NBT) by superoxide radicals generated during hydroxylamine hydrochloride auto-oxidation. Sodium carbonate 50 mM, 5 mL, EDTA 0.1 mM (pH 10.8) 0.1 mL, and 96 mM nitroblue tetrazolium 0.2 mL were combined for the SOD experiment. This assay mixture was supplemented with 0.2 mL of hydroxylamine hydrochloride and 0.05 mL of homogenized tissue supernatant. This causes hydroxylamine hydrochloride to auto-oxidize. Absorbance was measured in a UV-visible spectrophotometer at 560 nm for 2 min at 30-s intervals. SOD units per milligram of protein were used to compute and express the results (Akhtar et al., 2021).

###### 2.3.2.5.4 Reduced glutathione (GSH)

GSH is estimated using Ellman’s reagent (DTNB), which reacts with free sulfhydryl groups of glutathione to form a yellow-colored chromogen (TNB). 1 mL of homogenized tissue supernatant and sulfosalicylic acid (4% w/v) 1 mL were used for the GSH test. The mixture was incubated for 10-15 min at 4 °C. After getting a precipitate, the mixture was centrifuged at 12000 rpm for 10 min at 4 °C to produce a supernatant. By adding 0.2 mL of the supernatant to 0.4 mL of 0.01 M 5,5-dithiobis-(2-nitrobenzoic acid)-DTNB (pH 8.0) and 5.4 mL of 0.1 M phosphate-buffered saline (pH 7.4), a total volume of 6 mL was obtained. The observed result was a yellow color. Using a UV-visible spectrophotometer, absorbance was measured at 412 nm. To determine the results, the molar extinction coefficient of the chromophore (1.36 × 104 M^-1^ cm^-1^) was used, which is then represented as micromoles of GSH/mg protein (Akhtar et al., 2021).

###### 2.3.2.5.5 Catalase activity

Catalase activity is assessed by measuring the hydrogen peroxide decomposition. A 6 mL mixture of 0.1 mL of tissue supernatant, 0.05 M phosphate-buffered saline (pH 7.0) 5.8 mL, and 0.019 M hydrogen peroxide 1.0 mL was prepared. Using a UV-visible spectrophotometer, the absorbance was recorded at 240 nm for 2 minutes at 30-s intervals. The findings were computed and presented as micromoles of H_2_O_2_ decomposed/min/mg protein (Akhtar et al., 2021).

###### 2.3.2.5.6 Malondialdehyde (MDA)

MDA, a lipid peroxidation product, reacts with thiobarbituric acid to produce a pink-colored thiobarbituric acid reactive substance (TBARS). In this assay, 0.5 mL of tissue supernatant was mixed with Tris-HCl (0.5 mL), which was used as a test solution. After that, 10% TCA (1 mL) was added. The assay mixture was incubated on ice for 10-15 min. Centrifugation was carried out at 4 °C for 10 min at 10000 g. The supernatant was collected. 0.67% thiobarbituric acid (1.0 mL) was added to 1.0 mL of this supernatant. The mixture was heated in boiling water for 15-20 min, and then the samples were rapidly cooled to room temperature. The absorbance was measured at 532 nm. The molar extinction coefficient of the chromophore, 1.56 × 10^5^ M− 1 cm−1, was used in the calculations. The results were presented as MDA/mg protein, nmol (Akhtar et al., 2020).

###### 2.3.2.5.7 AChE and BChE activity

The assay is based on Ellman’s method, where AChE and BChE hydrolyze their respective substrates to produce thiocholine. Thiocholine reacts with DTNB to form a yellow-colored product. An indicator of cholinergic neuron loss is the amount of acetylcholinesterase and butyrylcholinesterase. The Ellman approach was used quantitatively to evaluate the brain’s acetylcholinesterase and butyrylcholinesterase levels. Supernatant 0.2 mL, 0.01 M sodium phosphate buffer (pH 8) 5 mL, and DTNB (Ellman reagent) 0.2 mL were included in the test mixture. Acetylthiocholine iodide 0.2 mL) and butyrylthiocholine iodide 0.2 mL were added to the respective test tubes to start the reaction. At 412 nm, absorbance was recorded using a spectrophotometer. The findings were reported as a percentage of the control and computed using the chromophore’s molar extinction coefficient (1.36 × 104 M−1 cm−1) (Kumar et al., 2008).

##### 2.3.2.6 Histopathology

The intact brain was separated and carefully cleansed with phosphate-buffered saline (PBS) to prevent damage in order to perform a histological investigation of the hippocampus. For fixation, brain tissues were immersed in tubes filled with a 10% formalin solution. The tubes were kept for 24 hours at room temperature. Embedding of the tissue was done by transferring the tissue from the final wax bath to a mould filled with molten paraffin wax. A microtome was used to cut thin sections of tissue blocks of 4 microns. The tissue sections were floated in a water bath at a temperature of 50°–52 °C and then taken on microscopic slides. The tissue slices were then dehydrated using higher gradient concentrations of an aqueous alcohol solution after being dewaxed. Hematoxylin and eosin dye were used to stain the slices. The coverslip was placed on the slide, and the LX-500 LED trinocular research microscope (Labomed) was used to observe, and images were taken with the MiaCam CMOS AR 6pro microscope camera connected to Image AR Pro software 9 (Akhtar et al., 2021). Histopathological changes were qualitatively assessed in brain sections based on neuronal degeneration/necrosis, neuronal loss, vacuolation, and gliosis. The severity of the observed changes was graded as nil (−), mild (+), moderate (++), or severe (+++).

### 2.4 Statistical analysis

GraphPad Prism version 8.4.3 (GraphPad Software, San Diego, CA, United States) was used to perform statistical analysis. Data were analyzed by one-way analysis of variance (ANOVA) and a mixed-effects model, which was expressed as mean ± SEM, followed by Tukey’s *post hoc* analysis. The significance level was considered as ^*^p < 0.05.

## 3 Results

### 3.1 *In silico* evaluation

#### 3.1.1 Prediction of ADME profile, drug-likeness, and biological spectra of trans-anethole

The compound shows no rule violations with one hydrogen bond acceptor, no hydrogen bond donors, and a MolLogP value of 3.27, with a molecular weight of 148.09 g/mol, and suggests favorable oral bioavailability, fully satisfying Lipinski’s criteria (molecular weight <500 g/mol, LogP <5, HBA ≤10, HBD ≤5). The Molsoft drug-likeness score was found to be −1.68.

Favorable physicochemical and pharmacokinetic characteristics were found by the trans-anethole drug-likeness analysis and *in silico* ADME. With two rotatable bonds and eleven heavy atoms, the compound exhibits moderate structural flexibility. With its very low topological polar surface area (TPSA) of 9.23 Å^2^, it has one hydrogen bond acceptor and no hydrogen bond donors, indicating excellent membrane permeability and possible blood-brain barrier penetration. A balanced hydrophilic-lipophilic profile appropriate for oral drug candidates is indicated by the consensus log P value of 2.79, which denotes moderate lipophilicity. Predictions for water solubility (log S ≈ −3.0) show moderate aqueous solubility. The compound was not predicted to be a P-glycoprotein substrate, but pharmacokinetic predictions showed high gastrointestinal absorption and BBB permeability. Trans-anethole showed inhibition of CYP1A2 but no inhibition of CYP2C9, CYP2C19, CYP2D6, or CYP3A4, indicating a relatively low risk of significant metabolic interactions. The compound demonstrated a bioavailability score of 0.55 and complied with Lipinski, Veber, and Egan rules without any infractions. Additionally, no Brenk structural alerts or PAINS were found, and the synthetic accessibility score of 1.47 denotes ease of synthesis. Overall, these results point to trans-anethole’s advantageous pharmacokinetic and drug-like qualities, bolstering its potential as a viable option for therapeutic uses involving the central nervous system (Supplementary Figure S1; Supplementary Table S1).

In the biological activity spectrum of trans-anethole, it is predicted to have possible immunomodulatory, apoptosis-regulating, and anti-inflammatory effects. Trans-anethole has multifunctional biological potential, including anti-inflammatory, antioxidant, neuroprotective, enzyme inhibitory, and immunoregulatory properties, according to the PASS study. This supports the drug’s therapeutic significance in inflammatory and neurodegenerative disorders (Supplementary Table S2).

#### 3.1.2 Identification of targets

In total, 450 targets were obtained for trans-anethole from various databases. 402 targets were identified from DIGEP-Pred, and 48 targets from the Comparative Toxicogenomics Database (CTD). 237 targets were obtained after merging and removing the duplicates. A total of 951 AD-related targets were obtained from various databases. 75 targets from the Therapeutic Target Database (TTD), 80 targets from Online Mendelian Inheritance in Man (OMIM), 75 targets from GeneCards, 506 targets from CTD, and 215 targets from DisGeNET. After merging and removing the duplicates from various databases, a total of 584 targets were obtained.

A total of 174 common targets were identified between the trans-anethole-associated and AD-associated target sets using a Venn diagram (Supplementary Figure S2).

#### 3.1.3 Gene set enrichment and gene ontology analysis

The protein-protein interaction (PPI) network shows a highly interconnected network with 171 nodes and 4,176 edges. A strong interaction density between the proteins was demonstrated by the average node degree of 48.8. The average local clustering coefficient (0.672) suggests significant functional clustering within the network. PPI enrichment p-value (<1.0 × 10^−16^) confirmed that the network is statistically significant and not formed by chance, and the observed interactions were significantly higher than the expected number of edges (1,596). The target proteins coordinated participation in related molecular pathways and strong biological connectivity are indicated by these findings (Supplementary Figure S3).

The KEGG pathway enrichment analyses and Gene Ontology (GO) offer a strong computational foundation for elucidating the biological significance of the experimental data. Molecular Function (MF), Cellular Component (CC), and Biological Process (BP) are standardized hierarchies that show the systemic functional changes taking place in the model. The enriched KEGG pathways, including AGE–RAGE signaling, apoptosis, and HIF-1 signaling, are closely linked to Alzheimer’s disease pathology. The AGE- RAGE interaction promotes oxidative stress and neuroinflammation, contributing to amyloid-β accumulation. HIF-1 signaling reflects hypoxia-related neuronal stress, while apoptosis pathways indicate progressive neuronal cell death. The significant FDR values confirm strong involvement of these pathways in AD-related mechanisms (Supplementary Table S3). Molecular Function shows the enrichment of enzyme binding, kinase binding, ubiquitin ligase binding, and transcription factor binding, suggesting dysregulation of signaling and protein modification processes. In Alzheimer’s disease, these functions are critical for tau phosphorylation, impaired proteostasis, and abnormal protein aggregation, which shows that the molecular functions are highly relevant to AD pathology. The cellular component shows enrichment in membrane rafts, mitochondrial outer membrane, and secretory vesicles, supporting involvement in amyloid precursor protein processing, mitochondrial dysfunction, and synaptic impairment. These cellular components are known to play central roles in oxidative damage and disrupted neuronal communication in Alzheimer’s disease. Biological Process shows strong enrichment in oxidative stress response, hypoxia response, and regulation of apoptosis, which directly aligns with core pathological features of Alzheimer’s disease (Supplementary Figure S4). A protein-pathway interaction network for the 17 pathways and 174 proteins was developed using Cytoscape (Figure 1). A multi-target and multi-pathway interaction profile linked to cognitive impairment was found in the compound-target-pathway network built for trans-anethole. The network showed that trans-anethole interacts with a wide range of protein targets (top 5 targets include AKT1, MAPK1, MAPK3, IKBKB, and MAPK8) and pathways (top 5: Alzheimer’s disease, the AGE-RAGE signaling pathway in diabetic complications, apoptosis, and the TNF signaling pathway), indicating potential computational association with oxidative stress, neuroinflammation, apoptosis, and synaptic regulation.

**FIGURE 1:**
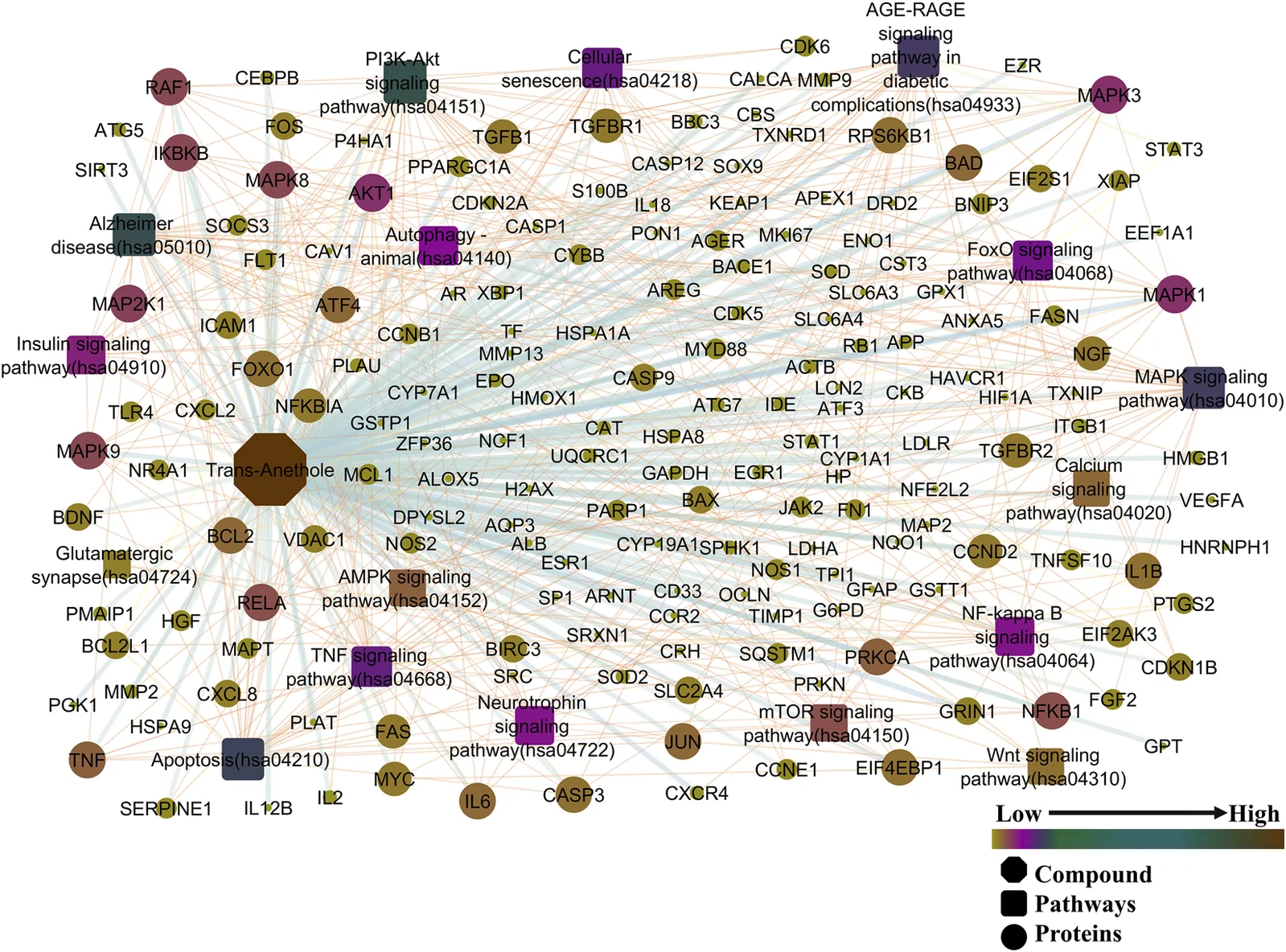
Protein-Pathway interaction of genes regulated by trans-anethole.

#### 3.1.4 Molecular docking

Docking was done for a total of 34 targets against trans-anethole and standard drugs using AutoDock Vina. In this, AKT1 showed a binding energy (−6.5 kcal/mol) with 5 π bond interactions (TRP80, VAL270, LYS268, LEU210) and 4 Van der Waals interactions (LEU264, SER205, ASN53, GLN79). IDE showed a binding energy of −6.4 kcal/mol with 1 hydrogen bond interaction (LEU204), 6 π bond interactions (TYR302, ALA479, LEU201, TYR314, LEU204), and 4 Van der Waals interactions (GLU205, ARG477, THR208, ILE304). CDK5 showed a binding energy of −6.4 kcal/mol with 8 π bond interactions (PHE80, CYS83, LEU133, ALA31, ALA143, LYS33, TYR15) and 6 Van der Waals interactions (GLU81, VAL64, ASP144, GLU51, PHE82, VAL18) (Figure 2; Table 1).

**FIGURE 2:**
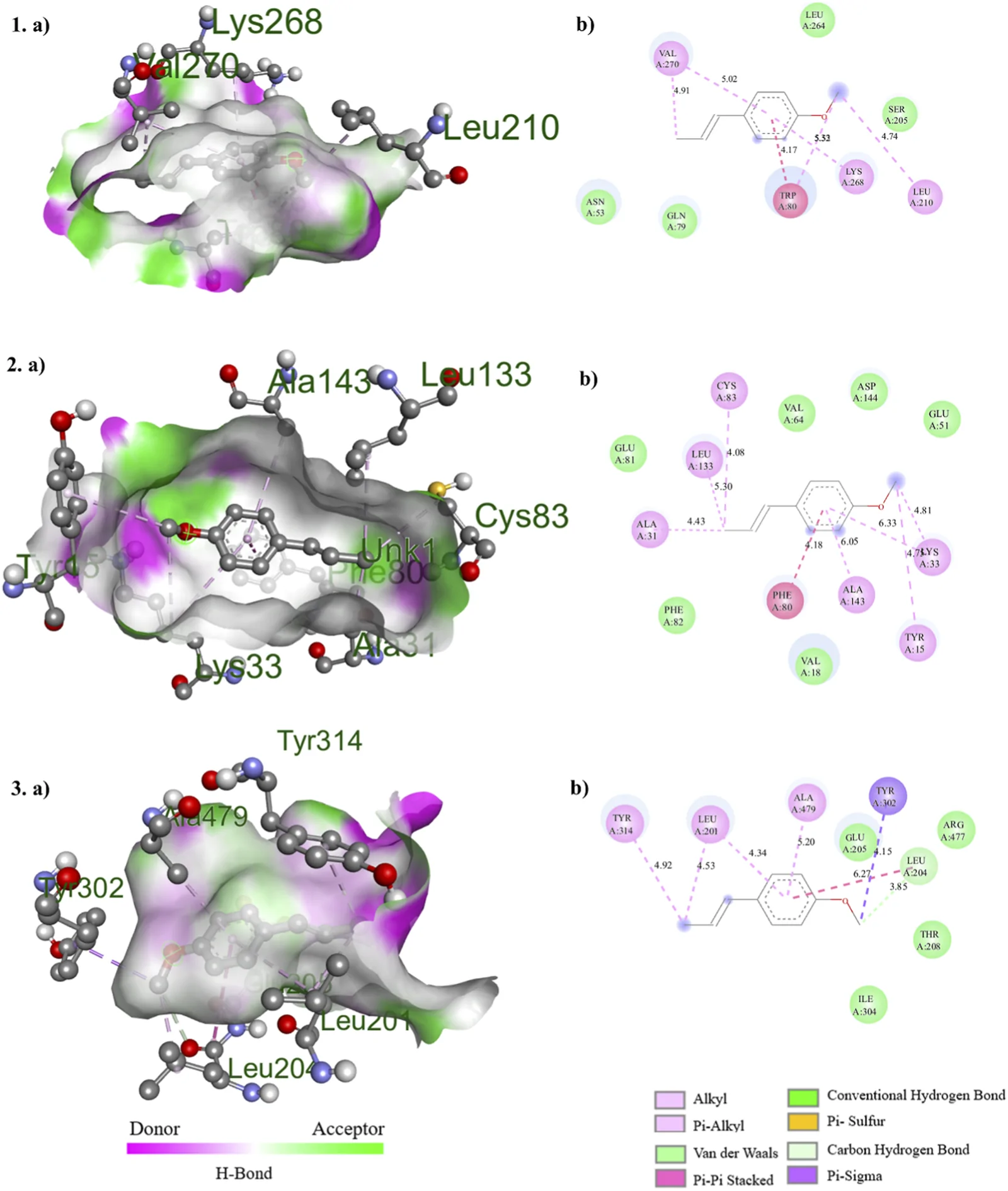
**(a)** 3D and **(b)** 2D interaction of (1) trans-anethole with AKT1; (2) trans-anethole with CDK5; (3) trans-anethole with IDE when docked in AutoDock Vina.

**TABLE 1:** Docking analysis of top 34 target proteins with trans-anethole using AutoDock Vina.

Target	BE	NHB	Residues	NPB	Residues	NVW	Residues
AGER	−6.2	0	NA	6	ARG344, PRO154, ALA63, TYR155, MET330, TRP340	4	GLU153, TYR341, TRP62, ASP65
AKT1	−6.5	0	NA	5	TRP80, VAL270, LYS268, LEU210	4	LEU264, SER205, ASN53, GLN79
APP	−4.1	0	NA	3	LEU165, LYS155	3	ASN164, SER159, GLU156
ATF4	−3.9	0	NA	4	TYR295, LYS299, ARG296, ARG300	2	GLN303, TRP84
BACE1	−5.8	1	TYR71	4	ASP32, TRY71, TRP76, VAL69	4	ILE118, PHE108, ASN37, SER35
BAD	−4.3	1	THR148	2	PHE151	3	TRP147, TRP155, GLN152
CASP3	−5.6	0	NA	8	PRO201, TYR197, TYR195, VAL266, VAL266, LYS137	5	ARG164, THR140, LEU136, ARG164, MET268
CASP9	−5.6	0	NA	2	PHE348, PHE351	5	THR347, ASP293, LYS398, GLN399, MET400
CDK5	−6.4	0	NA	8	PHE80, CYS83, LEU133, ALA31, ALA143, LYS33, TYR15	6	GLU81, VAL64, ASP144, GLU51, PHE82, VAL18
CYBB	−6.2	1	LEU69	5	PHE100, VAL14, PRO70, LEU76	6	ILE15, SER11, ARG96, GLN93, ASN97, LEU94
EIF2AK3	−6.1	0	NA	7	CYS891, PHE944, LYS893, LEU599, LEU890, ALA620, MET888	6	ARG892, VAL607, LYS622, ASP955, GLY954, VAL652
EIF2S1	−4.8	1	CYS199	6	LYS209, ILE205, VAL208, ALA198	5	TYR235, PRO232, VAL197, PRO233, GLY204
FAS	−5.1	1	ASP317	5	GLU179, VAL335, ARG328, LEU303, LYS299	7	SER325, GLN178, PHE327, ASN324, GLU337, GLN182, LEU306
GAPDH	−5.5	2	ASN34, ARG80	6	PRO191, PHE37, PRO36, PRO82, PHE102, THR99	5	GLY10, ASP35, GLU79, ASN9, ILE85
GRIN1	−5.3	1	ALA714	4	LYS483, VAL684, ALA714, ALA715	9	SER713, GLN686, LYS685, PHE484, ILE403, GLU712, GLY482, TRP731, GLN405
IKBKB	−6.2	1	CYS99	13	MET96, CYS99, LYS44, VAL29, ILE165, ALA42, VAL152, LEU21, TYR98	4	ASP166, VAL74, GLU97, GLY102
IL1B	−4.6	0	NA	5	ARG4, LEU6, ILE56, PHE46	1	SER5
IL6	−4.9	1	LYS171	6	ARG40, ARG168, LYS171, HIS164, LEU167	2	THR163, THR43
MAP2K1	−6.1	1	ASP594	8	TRP531, PHE583, CYS532, ALA481, LEU514, VAL471	4	ILE463, THR529, GLN530, GLY593
MAPK1	−4.9	1	ARG191	4	TRP,192, TYR113, TYR193, LYS151	3	PRO152, SER153, THR190
MAPK3	−6.2	1	THR85	4	ILE73, ARG84	7	LYS71, ASP184, GLY186, GLU88, TYR53, PRO75, TYR81
MAPK8	−5.6	0	NA	5	VAL40, MET111, VAL158, ALA53, ILE86	8	MET108, ILE32, GLY33, ASN114, SER34, GLU109, LEU110, LEU168
MAPK9	−6.2	1	MET111	7	MET108, LYS55, VAL40, ALA53, VAL158, ILE32, LEU168	3	ILE86, GLU109, LEU110
NFKB1	−5.2	2	VAL422, LEU420	4	LYS461, HIS468, LEU465	4	GLU460, GLN464, THR421, VAL428
NOS2	−6.3	0	NA	10	PHE619, LEU580, LEU684, LEU609, LEU612, LEU577, LEU615, ALA650	3	LEU645, LYS618, ASN617
PTGS2	−5.7	2	ARG44, ASN43	4	HIS39, PRO153, LEU152, LYS468	6	GLN461, GLU465, ARG469, GLY45, CYS41, GLN42
RAF1	−5.6	0	NA	7	CYS424, PHE475, TRP423, VAL363, ALA373	5	LYS375, THR421, LEU406, ILE355, GLY426
RELA	−5.6	1	LYS37	7	CYS38, LYS122, TYR36, LYS37, VAL121, ALA129	4	CYS120, GLN119, ASP125, GLN128
TNF	−4.4	2	SER136, LEU196	8	ILE231, LEU233, LEU133, TYR135, LEU133, TYR227	2	VAL199, TYR195
UQCRC1	−5.3	0	NA	4	PHE184, PHE344, ILE346	5	ARG180, ASP181, ASN185, ARG126, HIS188
VDAC1	−5.4	0	NA	3	LYS236, PHE18	5	GLY244, VAL237, ASN238, GLY220, ASN207
XBP1	−4.7	0	NA	4	TRP205, TRP208, CYS204	1	THR209
IDE	−6.4	1	LEU204	6	TYR302, ALA479, LEU201, TYR314, LEU204	4	GLU205, ARG477, THR208, ILE304
NOS1	−5.8	0	NA	5	VAL1215, LEU1048, HIS1212	8	HIS1045, ASN1044, ARG1222, SER1218, THR1106, HIS1213, THR1150, GLU1153

NA: not applicable; BE: Binding energy (kcal/mol); NHB: number of hydrogen bonds; NPB: Number of π bonds; NVW: number of van der waals forces.

#### 3.1.5 Molecular dynamics simulation and MMPBSA analysis

##### 3.1.5.1 Trans-anethole–AKT1 complex

The backbone RMSD during the first equilibration phase (0-20 ns) rapidly increased to 0.40-0.45 nm. For the rest of the 200 ns simulation, the system stayed steady with only slight variations around an average of about 0.40 nm. Indicating system equilibration, the RMSD of the complex increased rapidly during the first 5-20 ns, reaching 0.40-0.45 nm. After that, during the 200 ns simulation, it stayed steady with only slight variations (up to about 0.50 nm). Stable dynamics were indicated by the c-alpha RMSF values, which were primarily below 0.15 nm. Within the 117-123 region, residue 122 (1.14 nm) showed the largest fluctuation. Additional moderate peaks were found at the C-terminal region 441-444 (0.74 nm), residues 45-50 (0.27 nm), and 301-303 (0.36 nm). The RMSF complex values were less than 0.20 nm. At residue 122 (1.18 nm), the largest fluctuation was noted. The C-terminal 444 (0.79 nm), residues 45-49 (max 0.35 nm), 301-302 (0.41 nm), and 350 (0.33 nm) all had other notable peaks. The radius of gyration (Rg) of the backbone stayed constant throughout the 200 ns simulation, with the total Rg varying between approximately 2.19 and 2.29 nm. Slight variations in the axis-wise components, with RgX falling between 1.76 and 2.05 nm, RgY between 1.67 and 1.98 nm, and RgZ between 1.63 and 1.87 nm. The protein-ligand complex’s total radius of gyration stayed constant at 2.23-2.33 nm throughout 200 ns, suggesting that its compactness was maintained. Values along the axis displayed slight variations (RgX 2.08-1.81 nm, RgY 1.73-1.93 nm, and RgZ 1.66-1.90 nm) but no significant departures. The protein SASA first fluctuated (maximum 247.33 nm^2^), then gradually decreased and stabilized around 218-230 nm^2^, with a minimum of 216.51 nm^2^. During the 200 ns simulation, the protein-ligand complex’s SASA varied between approximately 215.38 and 246.27 nm^2^, stabilizing at approximately 220-230 nm^2^ in the subsequent phase. There were 0-2 hydrogen bonds between the protein and ligand during the 200 ns simulation. 0-1 hydrogen bonds were present for the majority of the time, with the occasional formation of two bonds. With a stable core and moderate interactions, the protein-ligand complex retained structural stability throughout the simulation, suggesting generally reliable binding (Figure 3, Supplementary File S1).

**FIGURE 3:**
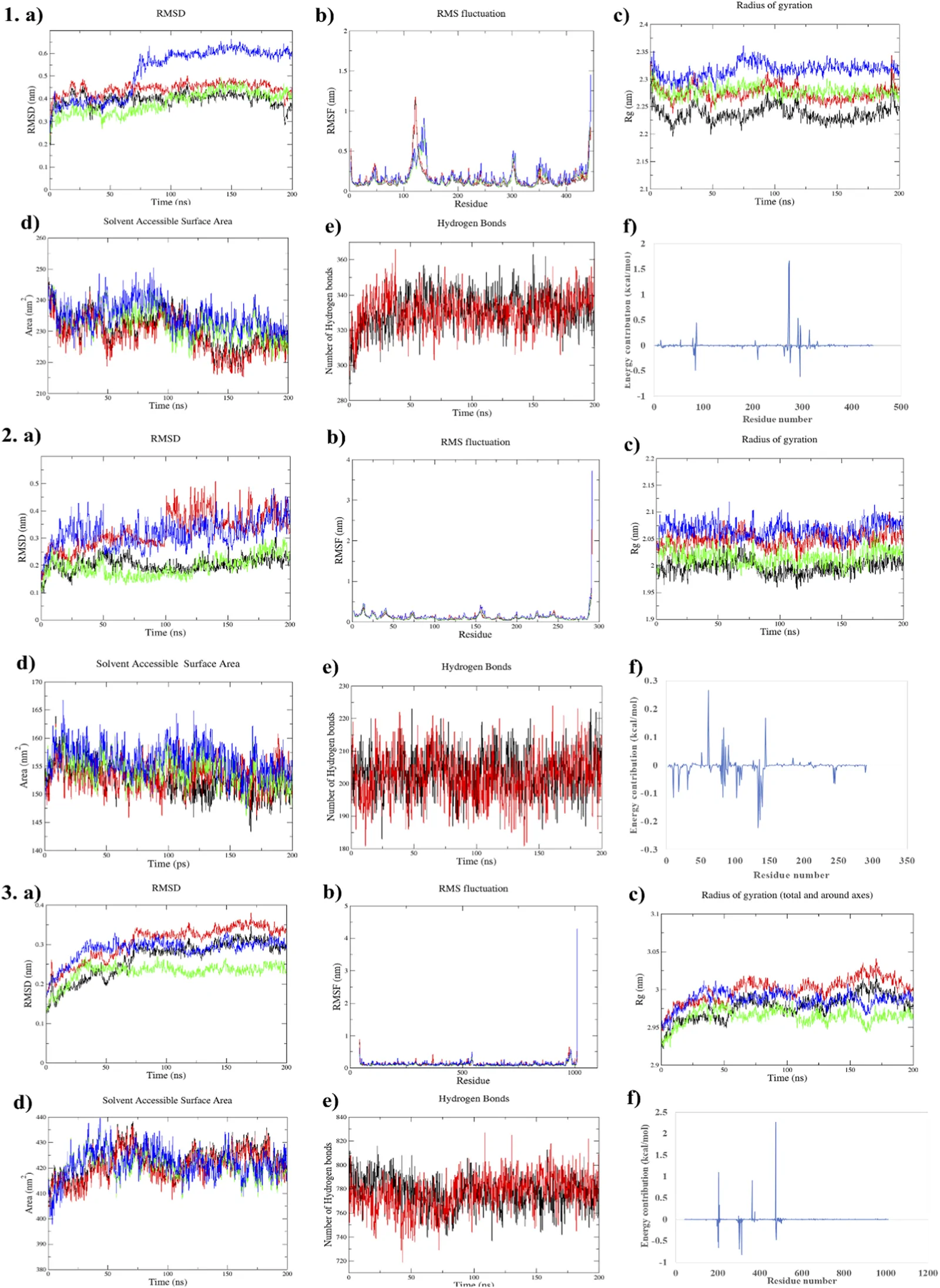
Stability described by trans-anethole with the target (1) AKT1; (2) CDK5; (3) IDE. **(a)** RMSD; **(b)** RMSF; **(c)** radius of gyration; **(d)** solvent accessible surface area (SASA); **(e)** number of hydrogen bonds between protein and ligand; **(f)** energy contribution for the trans-anethole–AKT1 complex. Black and green represent the backbone; red and blue represent the complex.

##### 3.1.5.2 Trans-anethole–CDK5 complex

During the first 5 ns, the RMSD backbone rose to about 0.20 nm, after which it stabilized between 0.17 and 0.25 nm, reaching a maximum of about 0.27 nm. The RMSD complex showed a transient peak of 0.51 nm at 140 ns before stabilizing at 0.33-0.38 nm, fluctuating between 0.25 and 0.32 nm, and increasing to 0.28 nm in 10 ns. The range of RMSF C-alpha was approximately 0.04–0.66 nm. With major peaks at 13-15 (0.35 nm) and 289-291 (max 0.66 nm), the majority of residues were stable (0.04-0.15 nm), suggesting flexibility primarily at terminal/loop regions. The RMSF of the complex varied between about 0.05 and 2.64 nm. With major peaks at 13-15 (0.37 nm), 154-156 (0.42 nm), and 289-292 (max 2.64 nm), the majority of residues were stable (0.06-0.20 nm), suggesting flexibility primarily at terminal regions. The Rg backbone stayed constant between 1.96 and 2.04 nm. Without significant structural expansion, axis-wise fluctuations were noted (X: 1.28-1.87 nm, Y: 1.30-1.85 nm, Z: 1.28-1.87 nm). The Rg complex stayed constant at 2.01-2.08 nm (0-80 ns). Stable compactness of the complex was indicated by axis-wise fluctuations (X: 1.32-1.91 nm, Y: 1.34-1.88 nm, Z: 1.32-1.91 nm) without structural expansion. This indicates that the complex remained reasonably compact during the simulation. The majority of SASA protein values fell between 148 and 156 nm^2^, with a range of approximately 143.43–163.89 nm^2^ (0-200 ns). The majority of SASA complex values fell between 148 and 158 nm^2^, with a range of 145.94–162.59 nm^2^ (0-200 ns). This indicates the complex’s structural integrity, as there was no significant expansion observed. Throughout the simulation, hydrogen bonds exhibited sporadic, weak interactions, primarily ranging from 0 to 1 and infrequently reaching 2 (Figure 3, Supplementary File S2).

##### 3.1.5.3 Trans-anethole–IDE complex

Over the course of the 200 ns simulation, the backbone RMSD increased from 0.0005 nm to 0.22 nm in 5 ns and then stabilized between 0.18 and 0.32 nm, indicating equilibrium and overall structural stability. The Protein_LIG complex RMSD grew from 0.0005 nm to approximately 0.18 nm by 1 ns, stabilized at 0.22-0.25 nm for 60 ns, and then progressively increased to a maximum of 0.356 nm at 118.6 ns, suggesting a generally stable structure with a small amount of flexibility later on. The RMSF c-alpha revealed moderate fluctuations at 42-50 (0.119-0.874 nm) and peaks at residues 976-1,010 (0.507-0.651 nm), while the majority of residues stayed stable (0.06-0.15 nm). High flexibility is indicated by the RMSF complex peaks at residues 42-45 and 976-1,012; the majority of other residues are stable (0.07-0.22 nm). suggesting overall structural stability. Over time, the radius of gyration (Rg) of the backbone stayed constant (2.95–2.99 nm) (Rg_X, Rg_Y, Rg_Z: 2.34-2.53, 2.34-2.42, 2.33-2.58 nm), suggesting some structural flexibility, particularly along the backbone’s Z-axis. The radius of gyration (Rg) of the complex stayed constant at 2.97-3.01 nm. Axial-specific values of Rg (X, Y, Z) (2.35-2.55, 2.35-2.45, 2.37-2.60 nm) showed slight variations primarily along Z. With variations between 410 and 430 nm^2^, the Solvent Accessible Surface Area (SASA) of the protein steadily increased from approximately 387 to approximately 433 nm^2^ over 200 ns. With variations between 410 and 430 nm^2^, the Solvent Accessible Surface Area (SASA) of the complex steadily increased from approximately 387 to approximately 433 nm^2^ over 170 ns, suggesting that the protein was exposed to the solvent. Rare and fleeting, hydrogen bonds were primarily zero, with sporadic ones of one or two at particular times (Figure 3, Supplementary File S3).

MMPBSA analysis was performed to estimate the binding free energy associated with the interaction of trans-anethole with AKT1, CDK5, and IDE using 100 snapshots extracted from the equilibrated MD trajectories. All calculated energies are expressed in kcal/mol and presented as mean ± SEM (n = 100). For the AKT1 complex, ΔVDWAALS, ΔEEL, ΔEPB, ΔENPOLAR, and ΔEDISPER were −22.14, −4.73, 20.80, −17.55, and 30.18 kcal/mol, respectively, resulting in a ΔGGAS of −26.87 kcal/mol and ΔGSOLV of 33.43 kcal/mol, with a ΔG_TOTAL of 6.56 kcal/mol. For CDK5, the corresponding values were −9.59, −1.03, 5.93, −7.78, and 12.72 kcal/mol, respectively, resulting in a ΔGGAS of −10.62 kcal/mol, ΔGSOLV of 10.87 kcal/mol, and ΔG_TOTAL of 0.25 kcal/mol. For IDE, ΔVDWAALS, ΔEEL, ΔEPB, ΔENPOLAR, and ΔEDISPER were −24.55, −1.79, 18.53, −18.13, and 30.56 kcal/mol, respectively, resulting in a ΔGGAS of −26.34 kcal/mol, ΔGSOLV of 30.96 kcal/mol, and ΔG_TOTAL of 4.63 kcal/mol. Overall, favorable van der Waals and gas-phase contributions were observed for all three complexes; however, these contributions were substantially counterbalanced by the positive solvation energy contributions, particularly ΔEPB and ΔGSOLV. Therefore, the MMPBSA results are considered supportive computational evidence of the energetic characteristics of the trans-anethole-target complexes rather than definitive evidence of binding affinity or binding stability (Table 2).

**TABLE 2:** MMPBSA analysis of trans-anethole–target complex.

Targets	ΔVDWAALS	ΔEEL	ΔEPB	ΔENPOLAR	ΔEDISPER	ΔGGAS	ΔGSOLV	ΔTOTAL
AKT1	−22.14	−4.73	20.8	−17.55	30.18	−26.87	33.43	6.56
CDK5	−9.59	−1.03	5.93	−7.78	12.72	−10.62	10.87	0.25
IDE	−24.55	−1.79	18.53	−18.13	30.56	−26.34	30.96	4.63

All the data are presented in mean ± SEM (n = 100), and the unit for each parameter is kcal/mol.

Where, Δ VDWAALS: van der Waals molecular mechanics energy; Δ EEL: electrostatic molecular mechanics energy; ΔEPB: polar contribution to the solvation energy; Δ ENPOLAR: Non-polar contribution of solute–solvent interactions to the solvation energy; Δ EDISPER: Non-polar contribution of attractive solute–solvent interactions to the solvation energy; Δ GGAS: total gas phase molecular mechanics energy; Δ GSOLV: total solvation energy; Δ GTotal: Total relative binding energy.

#### 3.1.6 Principal component analysis and free energy landscape

Controlled structural fluctuations during the simulation are indicated by the PCA projection (PC1 vs. PC2), which displays a well-clustered distribution of conformations with moderate dispersion. With PC1 accounting for 34.92% of the total variance and PC2 for 15.72%, it appears that the first two principal components captured the majority of important motions. Limited large-scale conformational transitions are indicated by the scree plot, which also confirms a sharp decline after the first few eigenvalues. Different areas of correlated (red) and anti-correlated (blue) motions were identified by DCCM analysis, indicating coordinated residue movements within functional domains. The AKT1 complex primarily occupied a stable conformational state during the simulation, as evidenced by the Gibbs free energy landscape’s dominant deep energy basin and sparse secondary minima (Figure 4).

**FIGURE 4:**
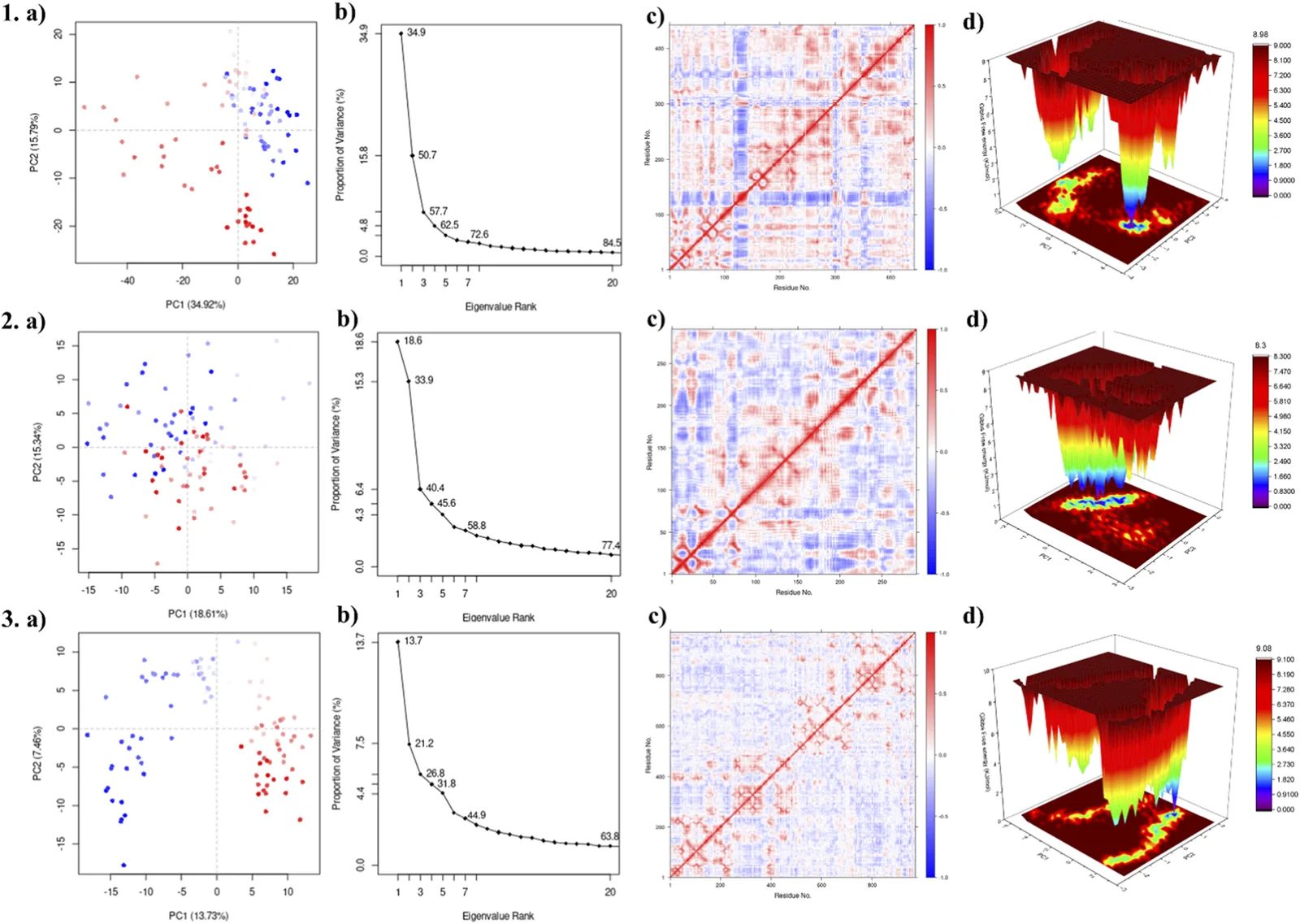
**(a)** Distribution of data points in a 2D space using principal components PC1 and PC2. **(b)** Percentage of variance explained by each principal component. **(c)** Dynamical cross-correlation matrix analysis. **(d)** Gibbs free energy landscape, for (1) AKT1; (2) CDK5; (3) IDE.

Although clustering remained compact overall, the PCA scatter plot for CDK5 showed relatively wider conformational sampling than AKT1. Distributed but controlled internal motions were indicated by PC1’s 18.61% explanation of variance and PC2’s 15.34%. The distribution of eigenvalues gradually decreased, indicating a moderate degree of flexibility. Dynamic communication between domains was suggested by the DCCM map’s balanced, correlated, and anti-correlated interactions throughout the protein structure. Indicating stable conformations with minor transitional states, the free energy landscape showed a principal low-energy basin with a few shallow neighboring minima. (Figure 4).

In the PCA plot, the IDE complex showed more pronounced separation, with discernible conformational transitions along PC1 (13.73%) and PC2 (7.46%). The conformational distribution indicated defined state transitions, even though the percentage contribution per component was lower than that of AKT1. Progressive eigenvalue decay was shown in the scree plot, indicating distributed essential dynamics. In addition to anti-correlated interactions that might support functional flexibility, DCCM analysis revealed prominent correlated motions within particular structural regions. Multiple energy basins with a single dominant minimum were shown in the Gibbs free energy landscape, indicating conformational flexibility while preserving overall structural stability (Figure 4).

### 3.2 Experimental pharmacology

#### 3.2.1 *In vitro* study

##### 3.2.1.1 Cell cytotoxicity using MTT assay

The MTT assay was used to assess the cytotoxic effects of donepezil and trans-anethole on BV2 cells after a 24-h exposure (1 nM–100 µM). The IC_25_ values of both compounds were calculated. The IC_25_ values indicate the concentration at which 25% of the cells undergo cytotoxicity. Cytotoxicity increased in both compounds in a concentration-dependent manner. Cytotoxicity following donepezil treatment ranged from 12.50% ± 1.96% at 1 nM to 48.79% ± 2.14% at 100 µM. Starting at 10 nM (33.00% ± 5.56%), there was a noticeable rise that continued at 100 nM (36.44% ± 5.81%), 1 µM (38.83% ± 4.75%), and 10 µM (41.64% ± 2.36%). Additionally, trans-anethole showed dose-dependent cytotoxicity, rising from 8.70% ± 6.21% at 1 nM to 42.99% ± 5.77% at 100 µM. The following intermediate concentrations showed progressive increases: 15.69% ± 4.44% (10 nM), 22.82% ± 0.69% (100 nM), 28.37% ± 4.88% (1 µM), and 38.37% ± 8.80% (10 µM). The IC_25_ values of donepezil and trans-anethole were found to be 6.485 nM and 168.9 nM after 24 h of treatment (Supplementary Figure S5).

##### 3.2.1.2 Antioxidant, AChE, and BChE potential of trans-anethole in BV2 cells

###### 3.2.1.2.1 Superoxide dismutase, reduced glutathione, and catalase levels

One of the main causes of neuronal degeneration in AD is oxidative stress. Antioxidant parameters showed a moderate improvement with donepezil. It shows dose-dependent SOD activity (0.39 ± 0.05, 0.27 ± 0.09, 0.23 ± 0.03, 0.29 ± 0.02, 0.83 ± 0.18, and 0.48 ± 0.02 U/mg protein at 1 nM, 10 nM, 100 nM, 1 μM, 10 μM, and 100 μM, respectively. Activity was increased at 10 µM (0.83 ± 0.18). Catalase activity peaked at 10 nM (3,749.24 ± 12.55 U/mg protein), whereas GSH levels were slightly elevated at lower concentrations, peaking at 0.1 µM (0.07 ± 0.02 µMol/mg protein). At higher concentrations, however, the antioxidant response seemed uneven. Trans-anethole demonstrated relatively greater antioxidant activity. At 10 µM (1.01 ± 0.09 U/mg protein), SOD levels were higher than those of donepezil at the same concentration. In contrast, GSH levels were also raised (0.17 ± 0.08, 0.32 ± 0.15, 0.22 ± 0.09, 0.26 ± 0.14, 0.08 ± 0.01, and 0.16 ± 0.08 µMol/mg protein). Catalase activity of trans-anethole was significantly higher (p < 0.05; vs. standard) than donepezil at most concentrations (3,423.61 ± 1.73, 6,326.92 ± 7.77, 6,311.53 ± 4.83, 5,879.76 ± 10.32, 5,525.55 ± 6.11, 4,582.87 ± 17.93 U/mg protein) (Figure 5).

**FIGURE 5:**
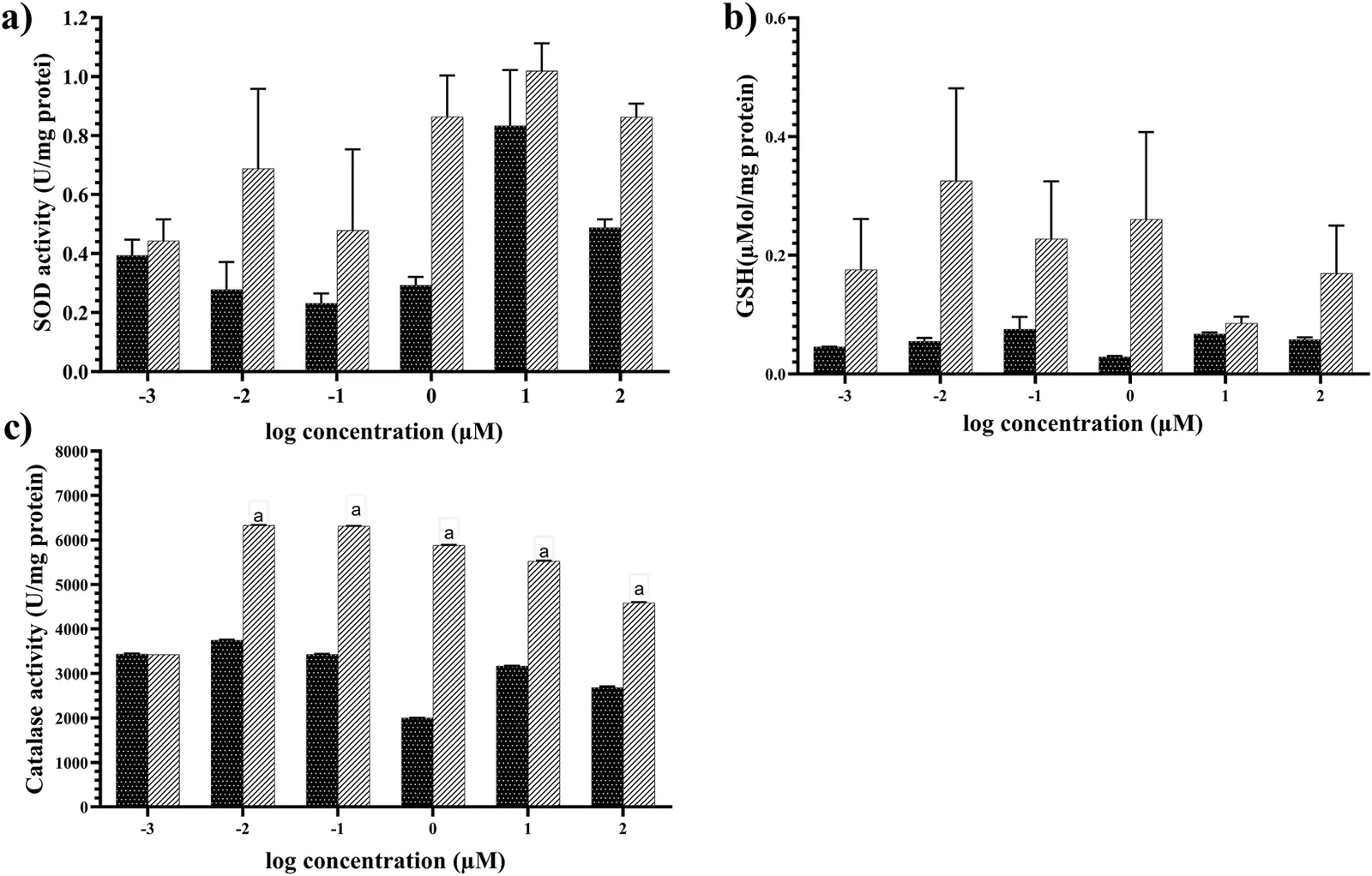
Assessment of antioxidant activity in BV2 cell line; **(a)** superoxide dismutase levels (U/mg protein); **(b)** reduced glutathione levels (µmol/mg protein); **(c)** catalase activity (U/mg protein). The data obtained were analyzed by one-way ANOVA followed by Tukey’s test for *post hoc* multiple comparisons using GraphPad Prism version 8.4.3. Values are expressed as mean ± SEM, (n = 3). ^a^p<0.05 is statistically significant compared to standard group.

###### 3.2.1.2.2 AChE and BChE activity

The effects of donepezil and trans-anethole on AChE and BChE enzyme activity were evaluated across a concentration range of 1 nM–100 µM. Donepezil inhibited AChE in a concentration-dependent manner (0.017 ± 0.0001, 0.019 ± 0.0001, 0.017 ± 0.0001, 0.010 ± 0.0001, 0.016 ± 0.0007, and 0.015 ± 0.0005 μmol/min/mg tissue); the highest suppression was seen at 1 µM (0.010 ± 0.0001). At higher concentrations (10-100 µM), enzyme activity stayed low, suggesting long-term inhibitory potential. This shows donepezil’s well-known high affinity for the catalytic active site of AChE; the significant decrease at 1 µM indicates the best possible enzyme-inhibitor interaction at this concentration. Whereas at all tested concentrations, trans-anethole showed relatively less AChE inhibition (0.019 ± 0.0005, 0.033 ± 0.0004, 0.033 ± 0.0009, 0.031 ± 0.0001, 0.028 ± 0.0006, and 0.081 ± 0.0262) compared to donepezil. A significant rise in enzyme activity was recorded at 100 µM (0.081 ± 0.0262), indicating decreased inhibitory efficacy at higher concentrations, despite slight decreases seen at lower doses.

With comparatively constant low activity values ranging from 0.014 to 0.019 (0.017 ± 0.0007, 0.019 ± 0.0003, 0.017 ± 0.0009, 0.016 ± 0.0058, 0.016 ± 0.0001, 0.014 ± 0.0012 μmol/min/mg tissue) across concentrations, donepezil demonstrated a moderate inhibition of BChE. This demonstrates that it is more selective for AChE than BChE. On the other hand, trans-anethole at lower concentrations showed only mild inhibition (0.023 ± 0.0052, 0.033 ± 0.0008, 0.032 ± 0.0007, 0.030 ± 0.0014, 0.043 ± 0.0147, and 0.088 ± 0.0386); however, at 10 µM (0.043 ± 0.0147) and 100 µM (0.088 ± 0.0386), BChE activity significantly increased, suggesting weak and non-selective inhibition at higher doses. Donepezil’s function as a symptomatic treatment for AD is supported by the cholinesterase inhibition data, which show that it successfully restores cholinergic balance by dramatically reducing AChE activity. On the other hand, the cholinesterase inhibitory capacity of trans-anethole is limited. These results support the pharmacological difference between the test compound and the standard drug, establishing trans-anethole as a potential adjunctive agent and donepezil as a major cholinergic modulator (Figure 6).

**FIGURE 6:**
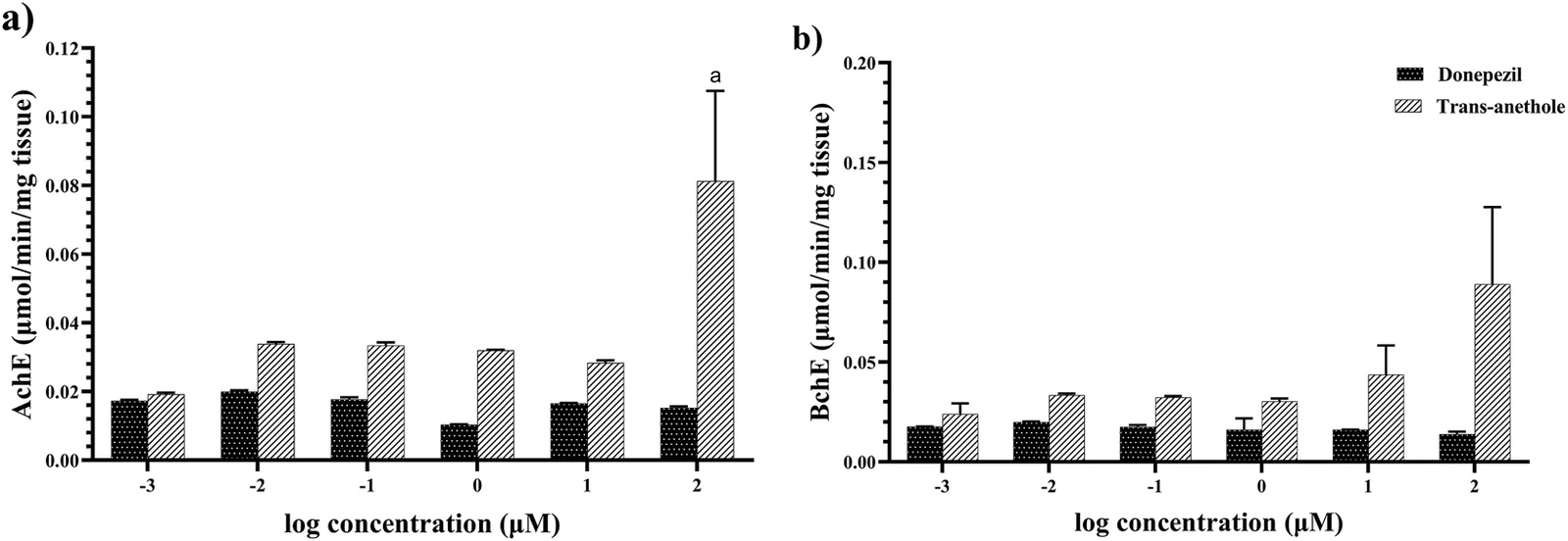
Assessment of AChE and BChE inhibition potential of trans-anethole and donepezil in BV2 cell lines. **(a)** AChE (μmol/min/mg tissue) inhibition assay. **(b)** BChE (μmol/min/mg tissue) inhibition assay. The data obtained were analyzed by one-way ANOVA followed by Tukey’s test for *post hoc* multiple comparisons using GraphPad Prism version 8.4.3. Values are expressed as mean ± SEM, (n = 3). ^a^p<0.05 is statistically significant compared to vehicle group.

#### 3.2.2 *In vivo* evaluation

##### 3.2.2.1 Behavioral studies

###### 3.2.2.1.1 Cohort I: open field test (OFT)

The anxiety-like behavior of rats was evaluated through the open field test on days 7, 14, 21, and 28 after STZ administration. Repeated behavioral measurements were analyzed using a mixed-effects model, with treatment as the between-subject factor and time as the within-subject factor. The analysis demonstrated a significant main effect of time (p = 0.0030) and treatment (p = 0.0015), whereas the treatment × time interaction was not significant (p = 0.8952). On day 7, in contrast to the vehicle control (1.60 ± 0.40), the disease control group showed a decrease in exploratory activity (0.20 ± 0.20), suggesting increased anxiety-like behavior. At the early stage, the standard and test-treated groups (100 mg/kg and 400 mg/kg) displayed similar low exploratory ratings, indicating symptoms associated with Alzheimer’s-like pathology. On Day 14, the disease control group showed decreased activity as compared to the normal group (0.40 ± 0.25). The standard (1.40 ± 0.40), test low-dose (1.20 ± 0.37), and test high-dose (1.20 ± 0.37) groups showed an improvement compared to the disease control. The behavioral ratings of treatment groups were close to the vehicle control group (1.80 ± 0.37), showing decreased anxiety-like behavior. On Day 21, the standard (1.60 ± 0.24) and high-dose test groups (1.40 ± 0.40) showed an increased number of central entries, whereas the disease control group (0.40 ± 0.40) continued to exhibit decreased exploratory behavior as compared to vehicle. A moderate improvement was observed in the low-dose group (1.20 ± 0.37). By day 28, the disease control group showed a significant decrease as compared to normal (0.40 ± 0.24; ^a^p<0.05 vs. vehicle); compared to the disease control group (0.40 ± 0.24), the standard (1.40 ± 0.51) and high-dose test groups (1.40 ± 0.24) showed consistent behavioral improvement. Though not as much as the high-dose group, the low-dose group also showed improvement (1.00 ± 0.31). Over the course of the experiment, the vehicle control continued to exhibit consistent exploratory behavior.

In this test, the disease control group exhibited reduced center entries compared with the vehicle control, indicating altered anxiety-like behavior following ICV-STZ administration. From Day 14 onwards, trans-anethole, particularly at the higher dose, increased center entries compared with the disease control group, suggesting an attenuation of anxiety-like behavioral changes. These OFT findings were interpreted as changes in anxiety-like behavior rather than as direct evidence of cognitive improvement (Figure 7).

**FIGURE 7:**
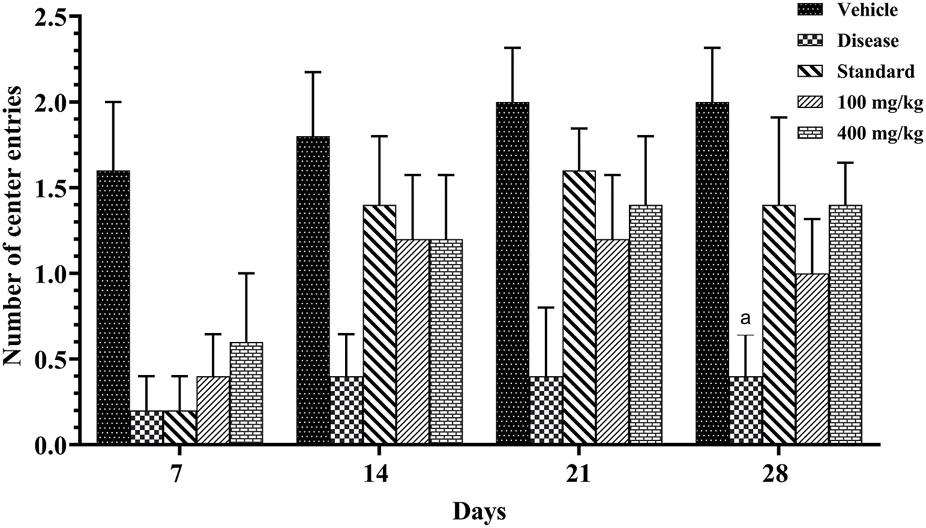
Measurement of anxiety-like behavior of rats among experimental groups on days 7, 14, 21, and 28 by open field test. The data obtained were analyzed by repeated behavioral measurements using a mixed-effects model, with treatment as the between-subject factor and time as the within- subject factor, using GraphPad Prism version 8.4.3. Values are expressed as mean ± SEM (n = 5). ^a^p<0.05 is statistically significant compared to vehicle control.

###### 3.2.2.1.2 Cohort II: spatial Y-maze memory test

Spontaneous alternation behavior was assessed on days 7, 14, 21, and 28 after STZ administration to evaluate spatial working memory. Repeated Y-maze measurements were analyzed using a mixed-effects model, with treatment as the between-subject factor and time as the within-subject factor. A significant main effect of time was observed (p = 0.0031), as well as a significant main effect of treatment (p = 0.0042). However, the time × treatment interaction was not significant (p = 0.7950), indicating that the pattern of treatment response did not differ significantly across the assessed time points. No missing observations were present. On day 7, spontaneous alternation was significantly lower in the disease control group (26.26% ± 2.37%, p < 0.05 vs. vehicle control) than in the vehicle control group (72.33% ± 8.43%). The standard group shows activity of 27.95 ± 10.59, whereas the low-dose (27.93% ± 9.50%) and high-dose (24.41% ± 6.32%) groups also displayed significantly lower values than the vehicle control (p < 0.05), suggesting impaired spatial working memory. On day 14, spontaneous alternation was still significantly lower in the disease control group (36.33% ± 4.41%; p < 0.05 vs. vehicle) than in the vehicle control group (67.57% ± 3.75%). While the low-dose (54.69% ± 12.26%) and high-dose (38.09% ± 16.51%) groups showed partial recovery, the standard treatment group showed improvement (56.2% ± 12.53%). By day 21, compared to vehicle controls (68.47% ± 4.57%), disease control animals showed impaired alternation behavior (44.48% ± 5.74%). The standard group improved even more (55.41% ± 16.12%), while the high-dose group showed a modest improvement (40.16% ± 6.35%, p < 0.05 vs. vehicle), and the low-dose group maintained a moderate recovery (54.55% ± 17.41%). On day 28, the disease control group’s spontaneous alternation rate (47.62% ± 5.12%) was lower than the vehicle control group’s (71.30% ± 5.28%). Compared to vehicle control levels, the standard treatment group demonstrated an improvement (67.59% ± 5.39%). While the high-dose group (40.07% ± 13.25%) showed relatively less recovery, the low-dose group (64.49% ± 8.96%) also showed marked improvement.

Spatial working memory deficits typical of Alzheimer’s-like pathology were confirmed by the disease control group’s persistent impaired spontaneous alternation behavior. This study shows that, over time, working memory was markedly restored after treatment with the standard drug. While the high dose of the test compound demonstrated relatively less efficacy, the low dose, in particular, produced progressive cognitive improvement. These results support the test compound’s possible neuroprotective role in the experimental Alzheimer’s model by showing that it improves spatial working memory deficits in a time-dependent manner (Figure 8).

**FIGURE 8:**
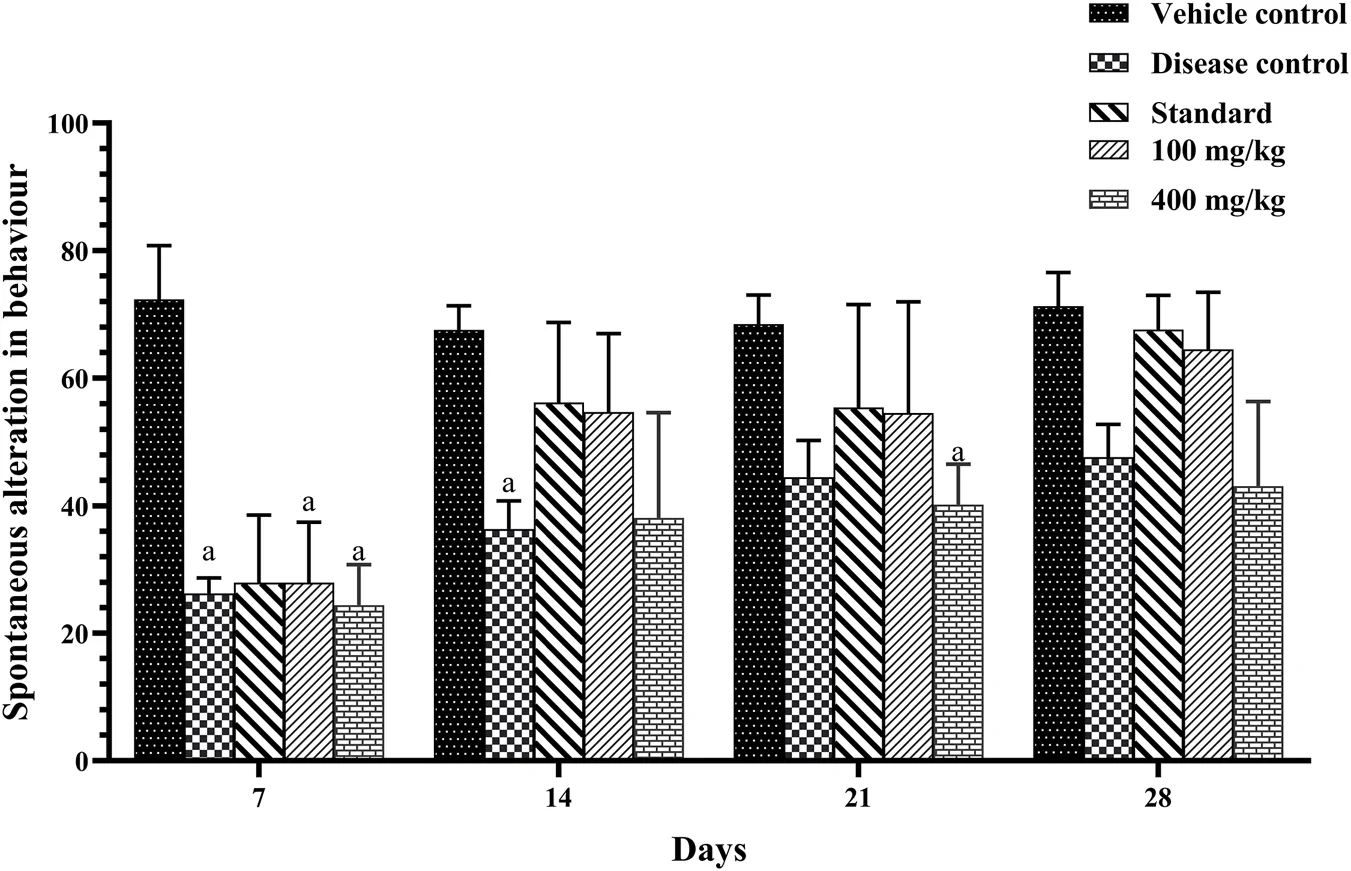
Measurement of spontaneous alternation behavior of rats among experimental groups on days 7, 14, 21, and 28 using the Y-maze test. Repeated Y-maze measurements were analyzed using a mixed-effects model, with treatment as the between-subject factor and time as the within-subject factor, using GraphPad Prism version 8.4.3. Values are expressed as mean ± SEM (n = 5). ^a^p<0.05 is statistically significant compared to the vehicle group.

###### 3.2.2.1.3 Cohort III: novel object recognition test

The discrimination index (DI) was assessed on days 7, 14, 21, and 28 after STZ administration to assess short-term recognition memory. Repeated measurements were analyzed using a mixed-effects model, with treatment as the between-subject factor and time as the within-subject factor. The analysis revealed a significant main effect of time (p = 0.0149) and a significant main effect of treatment (p = 0.0020). However, the time × treatment interaction was not significant (p = 0.5850), indicating that the pattern of treatment response did not differ significantly across the assessed time points. The vehicle control group demonstrated intact recognition memory on day 7 with a positive discrimination index (0.50 ± 0.07). The disease control group, on the other hand, showed a significant memory impairment (−0.34 ± 0.10, p < 0.05 vs. vehicle). At the same time point, the DI value of the standard group was −0.33 ± 0.32, and the high-dose group (−0.33 ± 0.17, p < 0.05 vs. vehicle) was also significantly less than that of the vehicle control, whereas the low-dose group displayed a milder reduction (−0.13 ± 0.23). By day 14, the vehicle control group showed 0.48 ± 0.12, while the disease control group showed impaired memory (−0.19 ± 0.21). The standard group improved, coming close to positive discrimination (0.16 ± 0.17). While the low-dose group stayed negative (−0.20 ± 0.37), the high-dose group showed a noticeable increase (0.37 ± 0.17). On day 21, in comparison to vehicle control (0.37 ± 0.17), the disease control group continued to show less discrimination (−0.14 ± 0.04). Positive discrimination indices demonstrated improvement in recognition memory in the standard (0.38 ± 0.17), low-dose (0.13 ± 0.24), and high-dose (0.13 ± 0.23) groups. On day 28, the disease control group’s DI remained lower (−0.15 ± 0.28), while the vehicle control group maintained a high DI (0.61 ± 0.03). The standard group showed recovery (0.50 ± 0.22), getting close to vehicle control values. When compared to previous time points, the low-dose (0.27 ± 0.18) and high-dose (0.28 ± 0.10) groups also demonstrated marked improvement. During the experiment, the disease control group continuously demonstrated compromised short-term recognition memory, mimicking the pathology of cognitive impairment. Over time, cognitive deficits were reversed by standard drug treatment. The positive results of the test compound suggest dose- and time-dependent improvement (Figure 9).

**FIGURE 9:**
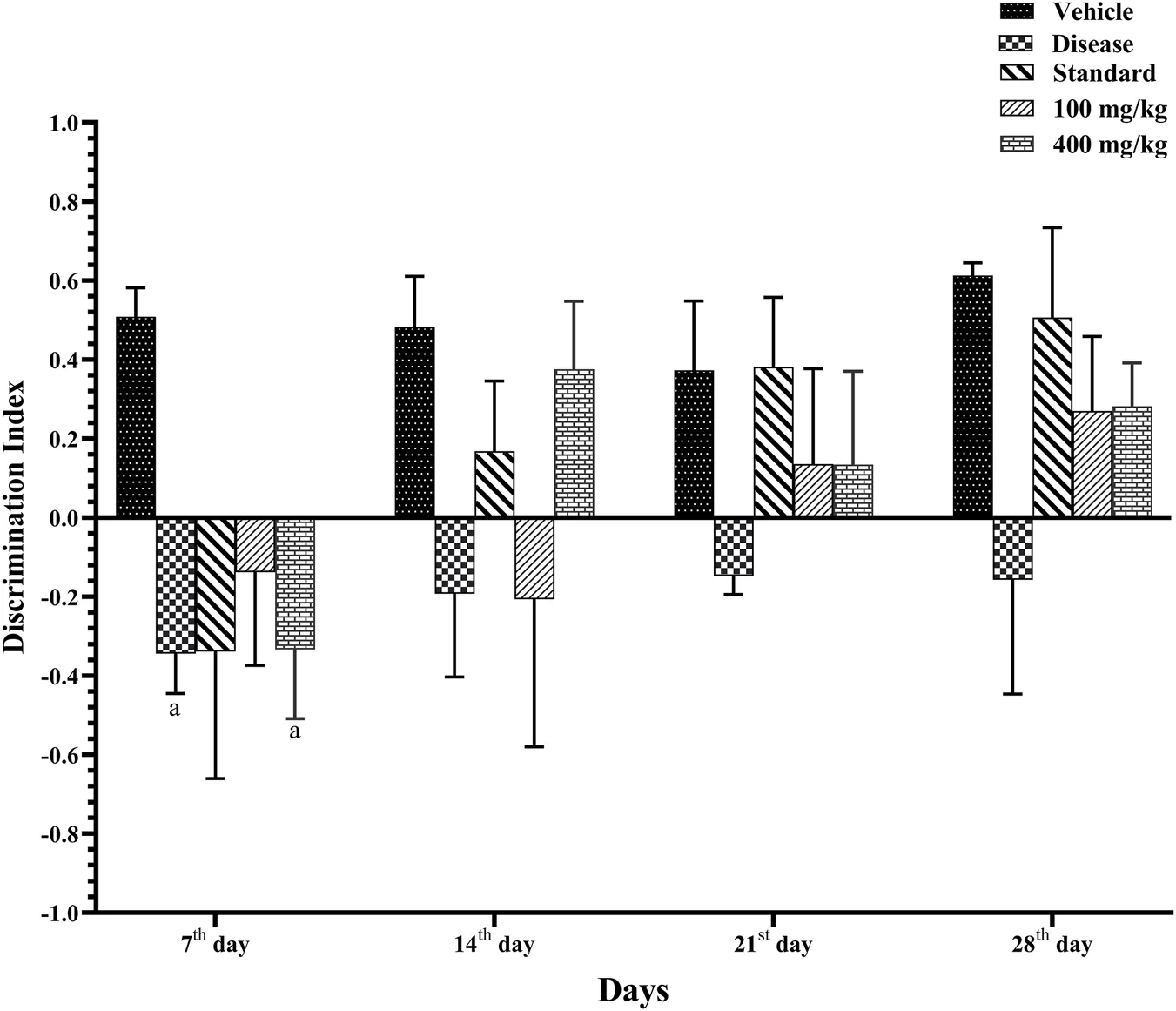
Measurement of the discrimination index among groups on days 7, 14, 21, and 28 using the novel object recognition test. To evaluate short-term recognition memory, the test phase was performed after 5 min following the sample phase. The data obtained were analyzed using repeated measurements by a mixed- effects model, with treatment as the between-subject factor and time as the within-subject factor, using GraphPad Prism version 8.4.3. Values are expressed as mean ± SEM (n = 5). ^a^p<0.05 is statistically significant compared to the vehicle group.

##### 3.2.2.2 Biochemical analysis

###### 3.2.2.2.1 Superoxide dismutase (SOD)

SOD levels in disease-control animals were significantly lower (19.12 ± 05.33 U/mg protein; p < 0.05 vs. vehicle control) as compared to the vehicle control animals (98.74 ± 09.19). Compared to the disease group, SOD levels of the standard drug (56.56 ± 11.45; p < 0.05 vs. disease) were significantly higher after treatment. In comparison to disease control, test compound administration also significantly increased SOD levels at both low doses (54.18 ± 07.96) and high doses (55.18 ± 08.24) (Figure 10).

**FIGURE 10:**
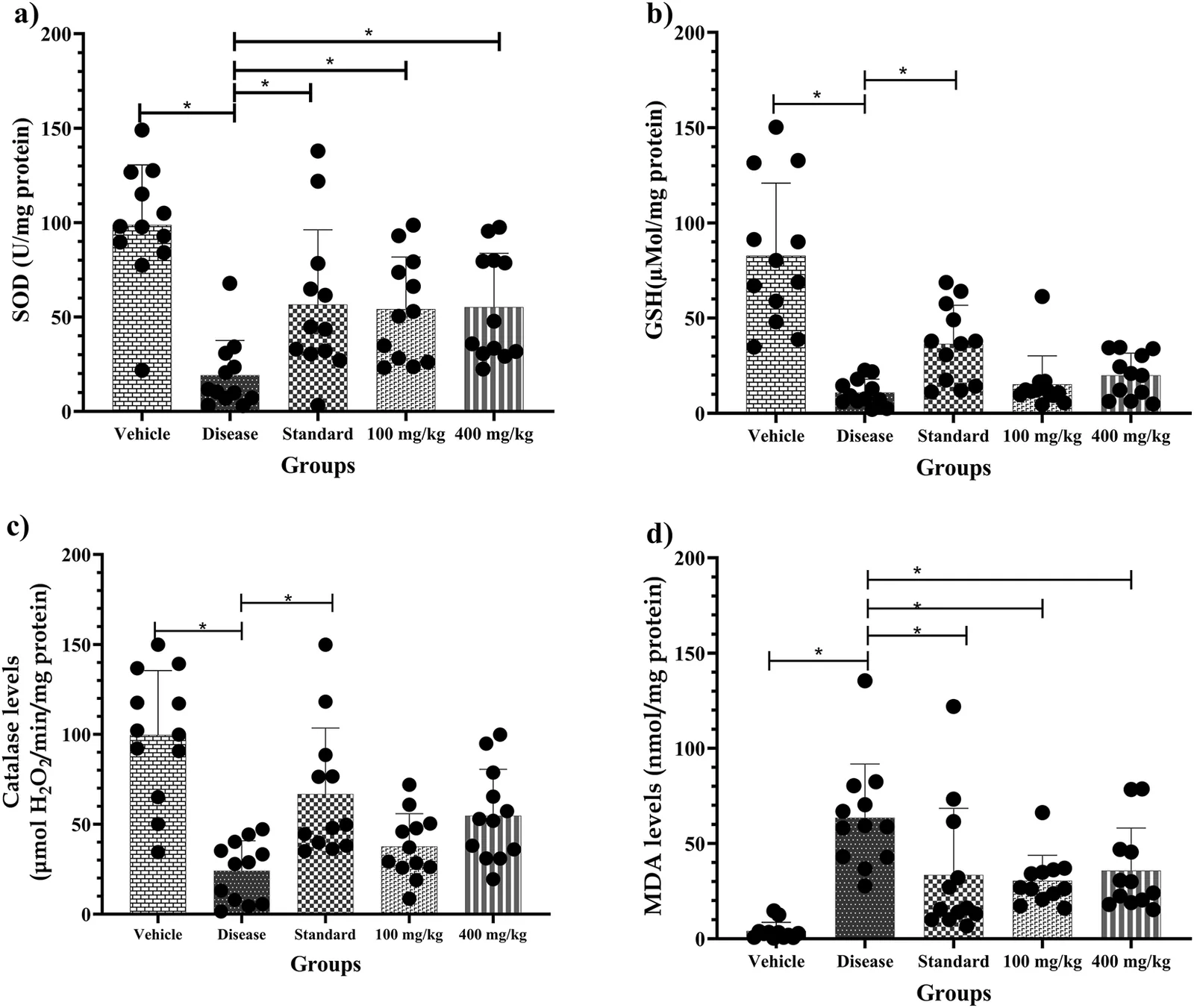
Assessment of antioxidant activity: **(a)** superoxide dismutase levels (U/mg protein); **(b)** reduced glutathione levels (µmol/mg protein); **(c)** catalase activity (U/mg protein); and **(d)** malondialdehyde levels (nmol/mg protein). The data obtained were analyzed by one-way ANOVA followed by Tukey’s test for *post hoc* multiple comparisons using GraphPad Prism version 8.4.3. Values are expressed as mean ± SEM (n = 12). *p < 0.05 represents statistical significance.

###### 3.2.2.2.2 Reduced glutathione (GSH)

GSH levels were significantly lower in the disease control group (10.89 ± 02.02 μmol/mg protein; p < 0.05 vs. vehicle control) than in the vehicle control group (82.69 ± 11.01). GSH levels were significantly restored after treatment with the standard medication (36.44 ± 05.85; p < 0.05 vs. disease). When compared to the disease group, the test compound raised GSH levels at both low and high doses (15.21 ± 04.31 and 19.95 ± 03.35) (Figure 10).

###### 3.2.2.2.3 Catalase activity

The disease control group’s catalase activity (24.14 ± 04.81U/mg protein; p < 0.05 vs. vehicle control) was significantly lower compared to the vehicle control group (99.58 ± 10.35). Catalase levels were significantly raised by standard treatment (66.75 ± 10.59; p < 0.05 vs. disease control). With a dose-dependent improvement, the test compound markedly increased catalase activity at both low and high doses (37.62 ± 05.27and 54.68 ± 07.45) (Figure 10).

###### 3.2.2.2.4 Malondialdehyde (MDA)

A measure of lipid peroxidation, MDA levels were significantly higher in the disease control group (63.48 ± 28.26 nmol/mg protein; p < 0.05 vs. vehicle control) than in the vehicle control group (03.97 ± 04.67). MDA levels were significantly lowered by standard treatment (33.45 ± 34.98; p < 0.05 vs. disease control). In comparison to disease control, the test compound significantly reduced MDA levels at both low and high doses (30.44 ± 13.35 and 35.75 ± 22.34; p < 0.05 vs. disease control) (Figure 10).

###### 3.2.2.2.5 AChE and BChE activity

AChE activity was significantly higher in the disease control group (13.77 ± 03.42 μmol/min/mg tissue; p < 0.05 vs. vehicle) than in the vehicle control group (01.38 ± 00.47). AChE activity was significantly lower in the standard drug group (02.97 ± 01.53; p < 0.05 vs. disease) than in the disease group. In comparison to the disease control, the test compound also showed a significant reduction in AChE activity at both low and high doses (03.86 ± 01.12 and 03.45 ± 01.17; p < 0.05 vs. disease). BChE activity was also significantly higher in the disease control group (11.12 ± 02.47 μmol/min/mg tissue; p < 0.05 vs. vehicle) than in the vehicle control group (01.87 ± 00.65). BChE levels were significantly lowered by standard treatment (02.90 ± 00.67; p < 0.05 vs. disease). When compared to disease control, the test compound’s administration significantly reduced BChE activity at both low and high doses (03.35 ± 00.30 and 02.66 ± 00.51; p < 0.05 vs. vehicle) (Figure 11).

**FIGURE 11:**
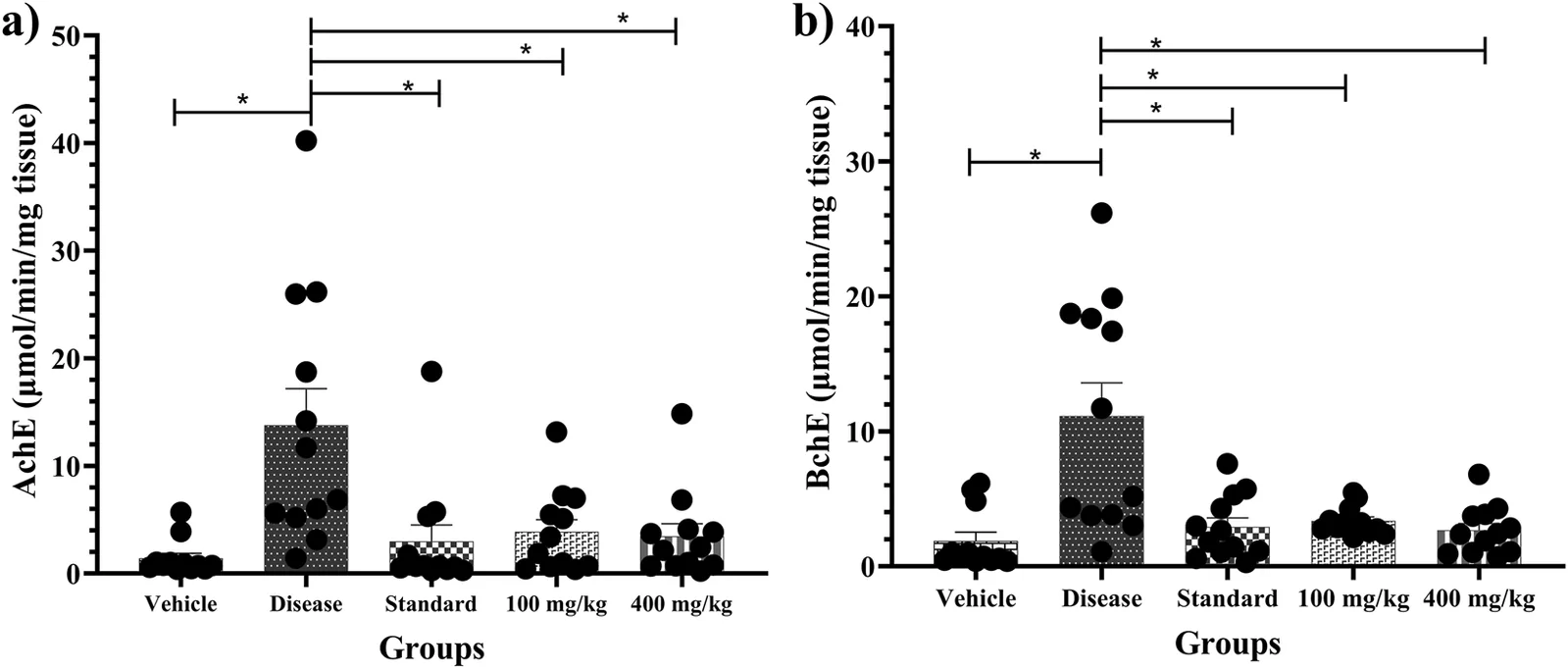
Assessment of **(a)** AChE (µmol/min/mg tissue) inhibition assay; **(b)** BChE (µmol/min/mg tissue) inhibition assay. The data obtained were analyzed by one-way ANOVA followed by Tukey’s test for *post hoc* multiple comparisons using GraphPad Prism version 8.4.3. Values are expressed as mean ± SEM (n = 12). *p < 0.05 represents statistical significance.

##### 3.2.2.3 Histopathology

Most of the neurons in the cerebrum & hippocampus are healthy, with pale and round vesicular nuclei, well-defined nuclear boundaries, and prominent nucleoli in the vehicle control group. There are no histological changes such as inflammation, degeneration, or necrosis. No change in tissue architecture. In the disease control, the dentate gyrus region of the hippocampus shows decreased neuronal cellularity (decreased neurons) with thinning of the granule cell layer consistent with neuronal loss. Some parts of the CA3 hippocampus show a reduction in neuronal density of the pyramidal cell layer. A few neurons of the CA1 region are necrotic, showing condensed eosinophilic cytoplasm with pyknotic nuclei. No histological changes are seen in the cerebrum or in tissue architecture. In the standard drug-treated group, increased neurons are seen in the CA3 region and dentate gyrus region of the hippocampus compared with the disease control. No histological changes were seen in the cerebrum, CA3, and CA1 regions of the hippocampus. No changes in tissue architecture. Compared with the disease control, there is an increase in the neurons of the dentate gyrus region and an absence of necrotic neurons in the hippocampus. The low-dose group shows a slight increase in neurons in the dentate gyrus and CA3 region of the hippocampus compared with the disease control slide. A few neurons of the CA3 region are necrotic, showing condensed eosinophilic cytoplasm with pyknotic nuclei. Very few degenerated neurons, which are basophilic, are seen in the cerebrum. No necrotic neurons in the CA1 region of the hippocampus. No changes in tissue architecture. Compared with the disease control, an increase in the neurons of the dentate gyrus & CA3 region of the hippocampus is seen in the low-dose group. The high-dose group showed an increased number of neurons in the CA3 and dentate gyrus regions of the hippocampus compared with the disease control slide. No necrotic neurons in the CA3 and CA1 regions of the hippocampus. No changes in tissue architecture. Compared with disease control, it increased the number of neurons in the dentate gyrus and CA3 region and an absence of necrotic neurons in the hippocampus. Overall, the histopathological findings suggest qualitative preservation of hippocampal neuronal morphology following trans-anethole treatment compared with the disease control group (Figure 12).

**FIGURE 12:**
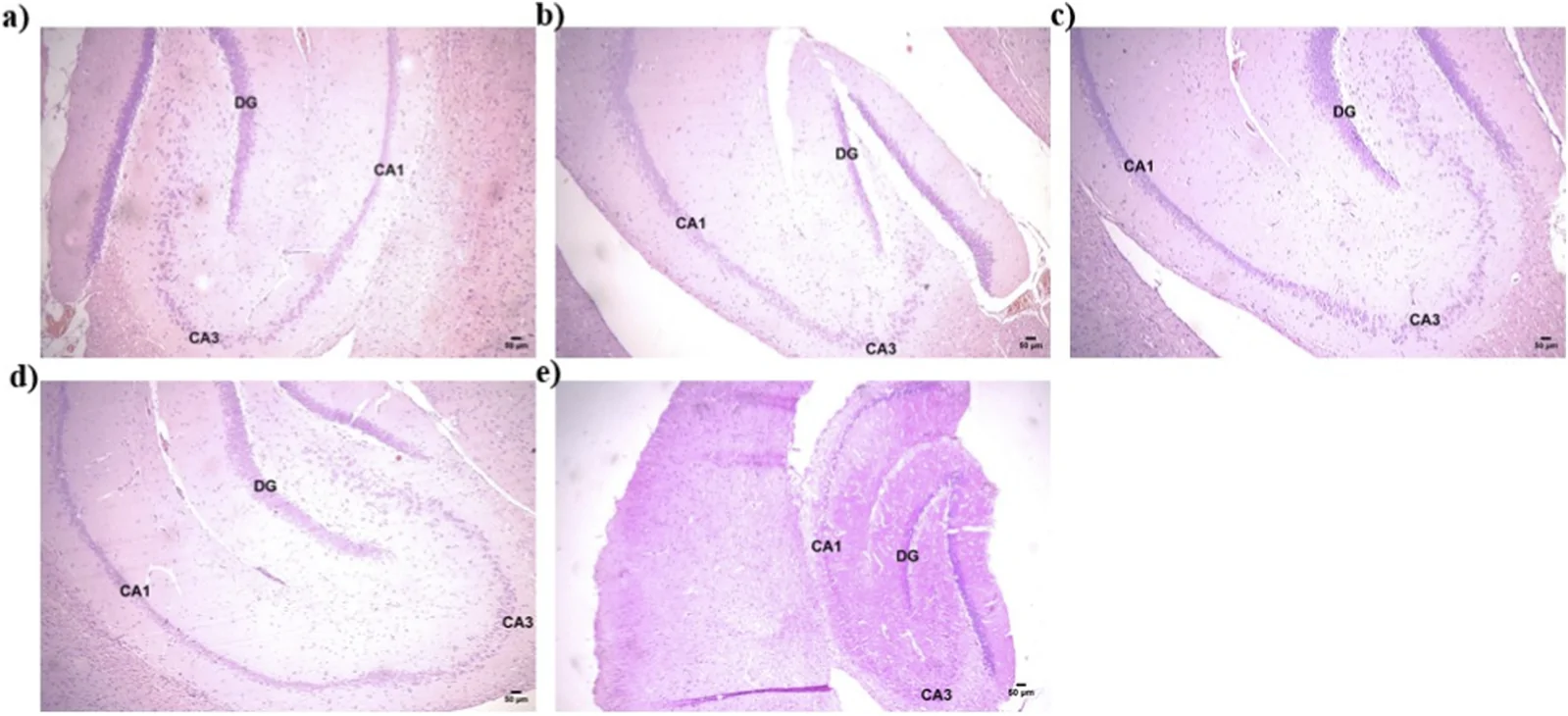
Histology of brain hippocampus at 40X. **(a)** Vehicle control; **(b)** disease control; **(c)** standard; **(d)** test (low dose); **(e)** test (high dose). DG: Dentate Gyrus; CA1: Cornu Ammonis 1; CA3: Cornu Ammonis 3.

## 4 Discussion

Alzheimer’s disease (AD), a multifactorial neurodegenerative disease, is typified by oxidative stress, cholinergic dysfunction, neuroinflammation, mitochondrial instability, impaired insulin signaling, and progressive cognitive impairment. The intracerebroventricular streptozotocin model used in this study reproduces several features associated with sporadic AD, including central insulin signaling abnormalities, oxidative stress, synaptic dysfunction, and cognitive impairment. However, ICV-STZ does not reproduce the full pathological complexity of human AD and should therefore be considered a model of selected AD-related features rather than a complete representation of the human disease. To clarify the neuroprotective potential and molecular mechanisms of trans-anethole, the current study methodically combined thorough *in silico* analysis, *in vitro* studies in BV2 cell lines, *in vivo* behavioral assessment, and biochemical evaluation in an ICV-STZ-induced Alzheimer’s model. Together, the results show that trans-anethole exhibits stable multitarget interactions with AD-associated proteins, restores antioxidant defense systems, and considerably improves cognitive deficits.

Multitarget therapeutic approaches are thought to be more effective than single-target strategies due to the multifactorial nature of AD. Several important proteins implicated in inflammatory, apoptotic, and neuronal survival pathways were discovered through target prediction and protein–protein interaction (PPI) network analysis. The primary putative molecular targets of trans-anethole identified through *in silico* study were AKT1, cyclin-dependent kinase 5 (CDK5), and insulin-degrading enzyme (IDE). However, their involvement was not experimentally validated in the present study and should therefore be considered predictive. Stable hydrophobic interactions and hydrogen bonds within the active site were among the favorable binding interactions between trans-anethole and specific AD-related targets that were shown by molecular docking studies, where AKT1, CDK5, and IDE showed the highest binding affinity. Static docking is unable to verify interaction stability in physiological settings. In order to verify complex stability, 200 ns molecular dynamics (MD) simulations were run. Stable backbone root mean square deviation (RMSD) profiles were found in the MD trajectories, suggesting that there was little conformational drift during simulation. While the active site residues stayed constant. Root mean square fluctuation (RMSF) analysis revealed restricted flexibility mainly in loop regions. Analysis of the solvent-accessible surface area (SASA) and radius of gyration (Rg) showed that structural compactness was maintained during the simulation, indicating that ligand binding did not cause destabilizing conformational changes.

Although solvation energies partially offset the total binding free energy, favorable van der Waals contributions to binding stability were found by molecular mechanics Poisson–Boltzmann surface area (MMPBSA) analysis. This indicates that the complexes did not display short-time, high-affinity interactions but rather a moderate, long-lasting interaction. In ligand-bound complexes, principal component analysis (PCA) showed limited large-scale conformational motion, and free energy landscape (FEL) analysis showed energetically stable conformational basins, which further supported the complexes’ structural stability. Collectively, these computational analyses suggest that trans-anetholecan adopts favorable and structurally stable poses within the selected protein targets under the simulated conditions. However, docking, molecular dynamics, PCA, FEL, and MMPBSA are predictive approaches and do not establish direct target engagement, binding affinity under physiological conditions, or downstream pharmacological activity in biological systems. In addition, MMPBSA provides an approximate computational estimate of binding free energy and is dependent on methodological assumptions. Therefore, the computational findings should be regarded as hypothesis-generating rather than definitive evidence of a multi-target mechanism. Experimental target-engagement studies will be required to determine whether AKT1, CDK5, and IDE or other predicted targets are directly modulated by trans-anethole.

According to the *in vitro* results, trans-anethole mainly modulates microglia and antioxidants to produce neuroprotective effects instead of directly inhibiting cholinesterase. Trans-anethole exhibits a favorable safety profile and a stronger redox regulatory capacity, supporting its potential role as an antioxidant candidate, even though donepezil continues to be superior in cholinergic restoration. The convergence of cellular antioxidant response and computational target prediction provides mechanistic evidence for trans-anethole as a multi-pathway modulator in AD. These results further motivated us to examine its neuroprotective effectiveness in an *in vivo* model in order to confirm its potential as a treatment under intricate physiological circumstances.

One of the main causes of AD’s hallmark, cognitive dysfunction, is the degeneration of cortical and hippocampal neurons. These deficiencies, which are consistent with disruption of insulin signaling and oxidative stress brought on by STZ, show hippocampal dysfunction and impaired synaptic plasticity. In the present study, the behavioral tests were interpreted according to their respective behavioral domains. The OFT was used to assess anxiety-like behavior and exploratory activity, whereas the Y-maze spontaneous alternation task was used to evaluate spatial working memory, and the NOR test was used to assess recognition memory. Compared with the vehicle group, the disease control group exhibited reduced center entries in the open field test, reduced spontaneous alternation in the Y-maze, and a negative discrimination index in the NOR test, indicating altered anxiety-like behavior, impaired spatial working memory, and impaired recognition memory, respectively. Trans-anethole treatment improved these behavioral measures compared with the disease control group. Importantly, the improvement observed in the OFT was interpreted as attenuation of anxiety-like behavior changes rather than as a direct indication of improved cognition. Similarly, Sahraei et al. reported that the STZ caused impairment of working memory with a significant decrease in alternative behavior when compared to control animals (40.67 ± 2.04 vs. 73.00 ± 1.88, p < 0.0001). Whereas oral administration of *Centella asiatica* improved STZ-induced working memory deficit (55.33 ± 3.34 and 57.17 ± 3.81 vs. 40.67 ± 2.04, p < 0.013, p < 0.004, respectively) (Sahraei et al., 2022; Tanvi et al., 2026). reported a significantly impaired recognition memory in the disease control group (−0.05 ± 0.02 at 2 h) compared to normal (p < 0.05), whereas the standard group improved the discrimination index (0.35 ± 0.02 at 2 h) compared to disease (p < 0.05), and diosgenin (0.07 ± 0.03 at 2 h) also showed a significant improvement in recognition memory (p < 0.05).

The Y-maze and NOR findings provide the primary behavioral evidence for improvement in spatial working memory and recognition memory, respectively. The stabilization of neuronal membranes, the reduction of oxidative stress, and possible modification of survival signaling pathways may all be responsible for these behavioral improvements. The Y-maze and NOR findings provide evidence of improved spatial working and recognition memory, respectively. However, performance in these tasks may also be influenced by exploratory drive, anxiety, motivation, and general activity levels. As locomotor activity was not separately quantified, subtle activity-related effects cannot be completely excluded. Thus, these findings should be interpreted as improved performance in memory-related tasks rather than definitive evidence of restored cognition. The observed improvements may reflect reduced oxidative stress and preservation of hippocampal neuronal function. As the integrity of the hippocampus becomes important for Y maze and NOR performance, the improvements we saw suggest that trans-anethole may protect hippocampal neurons.

By encouraging lipid peroxidation, mitochondrial dysfunction, protein oxidation, and DNA damage, oxidative stress plays a crucial role in the pathophysiology of AD. The decreased levels of superoxide dismutase, catalase, and reduced glutathione in the disease show a significant oxidative imbalance after STZ administration. This was confirmed by the elevated malondialdehyde levels in disease control animals as compared to vehicle control animals. Reduced membrane lipid damage is indicated by lower malondialdehyde levels; trans-anethole at both low and high doses showed a significant decrease in malondialdehyde levels. Enhanced endogenous antioxidant defense mechanisms are suggested by higher superoxide dismutase and catalase activity and reduced glutathione levels. A decrease in the lipid peroxidation level and restored antioxidant enzyme levels were observed in the treatment groups after trans-anethole treatment; this indicates strong antioxidant defense. As a phenylpropanoid, trans-anethole has the inherent ability to scavenge free radicals, potentially neutralizing reactive oxygen species and stopping oxidative cascades. Improved neuronal survival and cognitive function were probably facilitated by the restoration of redox balance. Therefore, the observed restoration of redox balance is consistent with a potential neuroprotective effect of trans-anethole; however, these findings do not by themselves establish a disease-specific or multitarget mechanism. The antioxidant effects may also represent a relatively nonspecific response to oxidative stress, and the contribution of the predicted molecular targets to these effects remains to be experimentally established.

The histopathological evaluation showed a qualitative preservation of hippocampal neuronal morphology following trans-anethole treatment, particularly in the dentate gyrus and CA1/CA3 regions. These findings provide morphological support for a potential neuroprotective effect of trans-anethole, which is consistent with the observed *in vitro* antioxidant activity and *in silico* predictions involving AKT1, IDE, and CDK5 pathways. However, as the histopathological assessment was qualitative, these findings should be interpreted cautiously and do not independently establish a reduction in cognitive impairment.

It is also important to note that the present study does not include data on pharmacokinetics and brain exposure. Trans-anethole was administered orally, but plasma and brain concentrations, bioavailability, brain penetration, and exposure-response relationships were not evaluated. Thus, it remains uncertain whether the administered doses produce sufficient concentrations of trans-anethole or its metabolites within the brain to engage the computationally predicted targets. Therefore, pharmacokinetics studies quantifying plasma and brain tissue levels will be important to establish the relationship between systemic administration, central exposure, and behavioral or biochemical effects.

The study mainly relied on biochemical results and computational predictions, while the predicted targets AKT1, CDK5, and IDE were not experimentally validated at the protein and gene levels using Western blot, RT-PCR, or immunohistochemistry. The network pharmacology provides computational predictions rather than direct evidence of pharmacological activity. Amyloid-β, amyloid precursor protein, total tau, and phosphorylated tau were not measured. The locomotor activity was not separately quantified in the OFT. Therefore, OFT findings were interpreted as anxiety-like behavior rather than direct cognitive measures. Only the BV2 microglial model was used in the study, which is not a neuronal cell line; therefore, our findings should not be interpreted as direct evidence of neuronal protection. Histopathological assessment was qualitative, and quantitative neuronal counts or blinded histological scoring was not performed. Further studies using activated microglial and neural models are needed to confirm the anti-neuroinflammatory and neuroprotective effects of trans-anethole. Tools such as Western blotting, qPCR, and immunohistochemistry can be used in order to confirm mechanistic pathways and to validate important targets. Additive or synergistic effects of trans-anethole with memantine, donepezil, and other Alzheimer’s disease mitigating drugs can be studied. Trans-anethole has a good safety profile, but long-term use validation requires dose escalation and chronic toxicity studies.

## 5 Conclusion

The current study used a sequential *in silico*, *in vitro,* and *in vivo* approach to understand the neuroprotective mechanism of trans-anethole in an Alzheimer’s model. The *in silico* findings identified AKT1, CDK5, and IDE as putative molecular targets of trans-anethole. Stable interactions were shown by molecular docking and molecular dynamics simulation. The *in vitro* study shows suppressed oxidative stress and modification of cholinesterase activity in BV2 microglial cells, suggesting the antioxidant and cholinesterase inhibition potential of trans-anethole. In the *in vivo* study, reduced anxiety-like behavior, enhanced spatial working memory, and improved recognition memory were observed. Behavioral paradigms such as the open field test, the Y maze, and novel object recognition tests were employed to evaluate the potential neuroprotective effects of trans-anethole by assessing anxiety-like behavior, spatial working memory, and recognition memory in the streptozotocin-induced Alzheimer’s model. The improved cognitive performance may be associated with reduced oxidative stress, restoration of antioxidant defense systems (superoxide dismutase, reduced glutathione, and catalase levels), decreased lipid peroxidation (malondialdehyde levels), and restored cholinergic activity. Histopathological findings confirm its neuroprotective role by demonstrating preservation of hippocampal neuronal architecture. Trans-anethole appears to exert its effect by restoring cholinergic activity, modulating microglial activation, and reducing oxidative stress. Collectively, these findings indicate that trans-anethole may represent a promising preclinical candidate to be further investigated in Alzheimer’s disease. The behavioral, biochemical, and computational data collectively support the potential neuroprotective properties of trans-anethole and provide a basis for further preclinical investigation of its efficacy, molecular mechanism, pharmacokinetic profile, and safety in cognitive impairment.

## Data Availability

The raw data supporting the conclusions of this article will be made available by the authors, without undue reservation.
