# Supplementary material for: Trans-anethole attenuates streptozotocin-induced cognitive dysfunction by modulating oxidative stress and cholinergic activity
Source: Front Pharmacol. 2026 Oct 16;17:1954658. doi: 10.3389/fphar.2026.1954658 (PMC13719536; doi:10.3389/fphar.2026.1954658)
Supplement: Supplementary file 1 [file Supplementaryfile1.docx]

**Supplementary figures and tables**

**Supplementary Figure 1.** Bioavailability radar plot for trans-anethole**.**

The hexagonal light pink background zone shows the "optimal" range for drug-likeness. The red shape indicates scores on each axis. The solid red polygon shows the compound’s actual values-points near the outer edge are ideal; inward deviations flag issues like poor solubility. **LIPO:** Lipophilicity; **SIZE:** Molecular size/weight; **POLAR:** Polarity; **INSOLU:** Insolubility; **INSATU:** Unsaturated bonds or rotatable bonds saturation; **FLEX:** Flexibility.

**Supplementary Figure 2.** Venn diagram of common targets between Alzheimer’s disease and trans-anethole

**Supplementary Figure 3.** Protein-protein interaction (PPI) network of common targets of Alzheimer’s disease and trans-anethole.

**Supplementary Figure 4.** Enrichment analysis. (A) KEGG pathway enrichment; (B) Gene Ontology analysis (molecular function); (C) Gene Ontology (cellular component); (D) tissue expression enrichment; (E) Gene Ontology analysis (biological process).

**Supplementary Figure 5.** Estimation of percentage cytotoxicity of donepezil and trans-anethole treated BV2 cells for 24 hours using MTT assay.

**Supplementary Tables**

**Supplementary Table 1.** The physicochemical properties, lipophilicity, water solubility, pharmacokinetics, druglikeness, and medicinal chemistry of trans-anethole.

| **Physicochemical Properties** | Heavy atoms | 11 |
| --- | --- | --- |
|  | Rotatable bonds | 2 |
|  | H-bond acceptors | 1 |
|  | H-bond donors | 0 |
|  | Molar Refractivity (MR) | 47.83 |
|  | TPSA | 9.23 |
| **Lipophilicity** | Log P_o/w_ (iLOGP) | 2.55 |
|  | Log P_o/w_ (XLOGP3) | 3.3 |
|  | Log P_o/w_ (WLOGP) | 2.62 |
|  | Log P_o/w_ (MLOGP) | 2.67 |
|  | Silicos-IT Log P_o/w_ | 2.79 |
|  | Consensus Log P_o/w_ | 2.79 |
| **Water Solubility** | Log S (ESOL) | -3.11 (1.15E-01 mg/ml) |
|  | Log S (Ali) | -3.17 (1.00E-01 mg/ml) |
|  | LogSw (Silicos-IT) | -2.98 (1.55E-01 mg/ml) |
| **Pharmacokinetics** | GI absorption | High |
|  | BBB permeant | Yes |
|  | Pgp substrate | No |
|  | CYP1A2 inhibitor | Yes |
|  | CYP2C19 inhibitor | No |
|  | CYP2C9 inhibitor | No |
|  | CYP2D6 inhibitor | No |
|  | CYP3A4 inhibitor | No |
|  | log Kp (cm/s) | -4.86 |
| **Druglikeness** | Lipinski violations | 0 |
|  | Ghose violations | 1 |
|  | Veber violations | 0 |
|  | Egan | 0 |
|  | Muegge | 2 |
|  | Bioavailability Score | 0.55 |
| **Medicinal chemistry** | PAINS alerts | 0 |
|  | Brenk alerts | 0 |
|  | Leadlikeness violations | 1 |
|  | Synthetic Accessibility | 1.47 |

**TPSA**: Topological Polar Surface Area; **BBB**: Blood-Brain Barrier; **Pgp**: P-glycoprotein; **CYP**: Cytochrome P450 enzymes; **log Kp**: Skin permeability coefficient; **PAINS**: Pan Assay Interference Compounds

**Supplementary Table 2.** The most relevant predicted activities of trans-anethole against Alzheimer’s disease.

| **Activity** | **Pa** | **Pi** |
| --- | --- | --- |
| JAK2 expression inhibitor | 0.886 | 0.004 |
| TNF expression inhibitor | 0.747 | 0.005 |
| MMP9 expression inhibitor | 0.726 | 0.005 |
| Caspase 3 stimulant | 0.705 | 0.011 |
| Interleukin antagonist | 0.663 | 0.005 |
| NOS2 expression inhibitor | 0.596 | 0.004 |
| HMOX1 expression enhancer | 0.587 | 0.021 |
| Free radical scavenger | 0.536 | 0.008 |
| Transcription factor NF kappa A inhibitor | 0.335 | 0.016 |
| Anti amyloidogenic | 0.323 | 0.069 |

**Supplementary Table 3.** KEGG pathway enriched genes.

| **term description (term ID)** | **OGC** | **BGC** | **Strength** | **FDR** | **Genes** |
| --- | --- | --- | --- | --- | --- |
| AGE-RAGE signaling pathway in diabetic complications(hsa04933) | 31 | 96 | 1.56 | 2.97E-34 | MAPK1, MMP2, TGFB1, SERPINE1, NFKB1, CDKN1B, EGR1, MAPK3, IL1B, STAT3, ICAM1, BAX, CXCL8, CASP3, FN1, TGFBR2, STAT1, JUN, AGER, CYBB, FOXO1, JAK2, MAPK8, BCL2, RELA, IL6, MAPK9, TNF, PRKCA, TGFBR1, AKT1 |
| Apoptosis(hsa04210) | 33 | 131 | 1.45 | 1.13E-33 | MAPK1, NFKBIA, NFKB1, TNFSF10, EIF2S1, MAPK3, BIRC3, BAX, MAP2K1, FOS, EIF2AK3, CASP3, PMAIP1, CASP9, ATF4, PARP1, MCL1, NGF, XIAP, JUN, BCL2L1, BAD, MAPK8, BCL2, RELA, MAPK9, TNF, RAF1, BBC3, IKBKB, AKT1, ACTB, FAS |
| PI3K-Akt signaling pathway(hsa04151) | 36 | 349 | 1.06 | 3.75E-25 | MAPK1, HGF, RPS6KB1, NFKB1, IL2, CDKN1B, EPO, CCND2, CCNE1, MAPK3, FGF2, CDK6, FLT1, MAP2K1, CASP9, ATF4, EIF4EBP1, FN1, MCL1, NGF, TLR4, BCL2L1, JAK2, BAD, AREG, ITGB1, BCL2, RELA, IL6, RAF1, PRKCA, BDNF, IKBKB, NR4A1, AKT1, MYC |
| TNF signaling pathway(hsa04668) | 25 | 111 | 1.4 | 1.08E-24 | MAPK1, NFKBIA, NFKB1, MAPK3, IL1B, BIRC3, ICAM1, MAP2K1, CEBPB, FOS, CASP3, SOCS3, ATF4, PTGS2, JUN, MMP9, MAPK8, RELA, IL6, MAPK9, TNF, CXCL2, IKBKB, AKT1, FAS |
| MAPK signaling pathway(hsa04010) | 32 | 286 | 1.1 | 1.97E-23 | MAPK1, TGFB1, HGF, NFKB1, MAPK3, IL1B, FGF2, FLT1, MAP2K1, FOS, CASP3, ATF4, MAPT, TGFBR2, NGF, JUN, MAPK8, AREG, RELA, MAPK9, TNF, RAF1, PRKCA, BDNF, IKBKB, HSPA8, NR4A1, TGFBR1, AKT1, MYC, MYD88, FAS |
| Cellular senescence(hsa04218) | 24 | 150 | 1.25 | 5.75E-21 | MAPK1, TGFB1, SERPINE1, NFKB1, CCNB1, CCND2, CCNE1, MAPK3, CDK6, RB1, MAP2K1, CXCL8, EIF4EBP1, TGFBR2, FOXO1, SQSTM1, VDAC1, RELA, IL6, RAF1, CDKN2A, TGFBR1, AKT1, MYC |
| FoxO signaling pathway(hsa04068) | 22 | 126 | 1.29 | 5.58E-20 | MAPK1, TGFB1, CDKN1B, CAT, TNFSF10, CCNB1, CCND2, MAPK3, STAT3, MAP2K1, SLC2A4, TGFBR2, BNIP3, FOXO1, MAPK8, IL6, MAPK9, RAF1, IKBKB, SOD2, TGFBR1, AKT1 |
| NF-kappa B signaling pathway(hsa04064) | 18 | 101 | 1.3 | 1.28E-16 | NFKBIA, NFKB1, IL1B, BIRC3, ICAM1, CXCL8, PARP1, PTGS2, XIAP, PLAU, TLR4, BCL2L1, BCL2, RELA, TNF, CXCL2, IKBKB, MYD88 |
| Autophagy - animal(hsa04140) | 19 | 131 | 1.21 | 4.69E-16 | MAPK1, RPS6KB1, EIF2S1, MAPK3, MAP2K1, EIF2AK3, HMGB1, ATG7, BNIP3, ATG5, BCL2L1, SQSTM1, BAD, MAPK8, BCL2, MAPK9, RAF1, HIF1A, AKT1 |
| Neurotrophin signaling pathway(hsa04722) | 18 | 112 | 1.25 | 6.15E-16 | MAPK1, NFKBIA, NFKB1, MAPK3, BAX, MAP2K1, ATF4, NGF, JUN, BAD, MAPK8, BCL2, RELA, MAPK9, RAF1, BDNF, IKBKB, AKT1 |
| Insulin signaling pathway(hsa04910) | 16 | 132 | 1.13 | 1.31E-12 | MAPK1, RPS6KB1, MAPK3, PPARGC1A, MAP2K1, FASN, SLC2A4, SOCS3, EIF4EBP1, FOXO1, BAD, MAPK8, MAPK9, RAF1, IKBKB, AKT1 |
| mTOR signaling pathway(hsa04150) | 10 | 150 | 0.87 | 4.54E-06 | MAPK1, RPS6KB1, MAPK3, MAP2K1, EIF4EBP1, TNF, RAF1, PRKCA, IKBKB, AKT1 |
| AMPK signaling pathway(hsa04152) | 8 | 120 | 0.87 | 4.72E-05 | RPS6KB1, PPARGC1A, FASN, SLC2A4, EIF4EBP1, SCD, FOXO1, AKT1 |
| Calcium signaling pathway(hsa04020) | 7 | 191 | 0.61 | 0.004 | SPHK1, NOS2, GRIN1, VDAC1, CXCR4, PRKCA, NOS1 |
| Glutamatergic synapse(hsa04724) | 4 | 112 | 0.6 | 0.0363 | MAPK1, MAPK3, GRIN1, PRKCA |
| Wnt signaling pathway(hsa04310) | 6 | 154 | 0.64 | 0.006 | CCND2, JUN, MAPK8, MAPK9, PRKCA, MYC |
| Alzheimer disease(hsa05010) | 35 | 354 | 1.04 | 5.45E-24 | UQCRC1, MAPK1, XBP1, NFKB1, EIF2S1, MAPK3, IL1B, IDE, APP, MAP2K1, EIF2AK3, CASP3, BACE1, NOS2, CASP9, ATF4, MAPT, PTGS2, GRIN1, AGER, CYBB, BAD, VDAC1, MAPK8, GAPDH, RELA, IL6, MAPK9, TNF, RAF1, CDK5, IKBKB, AKT1, NOS1, FAS |

**OGC:** Observed gene count; **BGC:** Background gene count; **FDR:** False discovery rate
